# Nanozymes for Liver Disease Therapy: Advances in Catalytic Activity, Targeting Strategies, and Clinical Translation

**DOI:** 10.1002/advs.74590

**Published:** 2026-02-28

**Authors:** Xiandi Meng, Ge Zhu, Siyu Sun, Wenbo Yao, Yuning Zhang, Yong‐Guang Yang, Tianmeng Sun

**Affiliations:** ^1^ Key Laboratory of Organ Regeneration and Transplantation of Ministry of Education Institute of Immunology The First Hospital Jilin University Changchun Jilin China; ^2^ National‐local Joint Engineering Laboratory of Animal Models for Human Diseases Changchun Jilin China; ^3^ Cancer Center The First Hospital of Jilin University Changchun Jilin China; ^4^ International Center of Future Science Jilin University Changchun Jilin China; ^5^ State Key Laboratory of Supramolecular Structure and Materials Jilin University Changchun Jilin China

**Keywords:** hydrolases, liver disease, nanozymes, redox reaction, ROS regulation

## Abstract

Natural and artificial enzymes have emerged as promising candidates for biomedical applications, possessing the potential to address redox imbalances and metabolic disorders in liver diseases. However, their clinical translation remains limited by challenges in biocompatibility, targeted delivery, dose‐response balance, and long‐term stability. Nanozymes, novel nanomaterials with potent enzyme‐like catalytic activity, exhibit promising capacities to overcome these challenges. In this review, we discuss nanozymes applied to liver diseases, including metabolic disorders, tissue repair, hepatic tumors, and viral infections. Under physiological and pathological liver conditions, diverse targeting strategies can be applied for the precise delivery of nanozymes. We demonstrate the fundamental mechanisms of nanozymes in scavenging reactive oxygen species (ROS), alleviating oxidative stress, and remodeling the immune microenvironment. The structural features, including size, morphology, composition, and surface modifications of nanozymes, critically influence catalytic performance and targeting efficiency at disease sites. These design criteria improve catalytic activity and reduce drug‐induced toxicity, thereby achieving a balance between dosage and therapeutic effects. We highlight the Artificial Intelligence (AI)‐driven revolution, in which machine learning contributes to predicting catalytic activity, optimizing structural design, and enabling intelligent applications of nanozymes. Finally, we outline the potential strategies and the limitations of nanozymes for clinical translation in liver disease therapy.

## Graphical abstract

Nanozymes, as enzyme‐mimicking nanomaterials, exhibit unique catalytic properties for the treatment of liver diseases. By regulating redox homeostasis, modulating immune responses, and enabling targeted delivery, nanozymes overcome the limitations of natural enzymes. Machine learning further accelerates their design, thereby supporting clinical translation in metabolic disorders, liver injury, hepatic tumors, and viral infections.

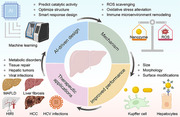

## 1 Introduction

Enzymes serve as powerful biocatalysts in most biological processes and are primarily composed of functional proteins along with a small number of catalytic RNA molecules. However, the stability, production cost, and storage requirements of natural enzymes limit their broad application [1]. Nanozymes are nanomaterials with the ability to mimic enzyme‐like catalytic activity. The term “nanozyme” was first introduced by Scrimin and colleagues in 2004, when they demonstrated that gold nanoparticles (AuNPs) exhibited unexpected ribonuclease‐like characteristics [2]. A significant milestone emerged with the 2007 study by Yan and coworkers, who discovered that iron oxide nanoparticles (Fe_3_O_4_) displayed remarkable peroxidase (POD)‐like activity [3]. Since then, a great number of studies on nanomaterial‐based artificial enzymes have been developed [4, 5]. After 2021, an updated definition was proposed: “nanomaterials that catalyze the conversion of enzyme substrates to products and follow enzymatic kinetics (e.g., Michaelis–Menten) under physiologically relevant conditions, even though the molecular mechanisms of the reactions could be different between nanozymes and the corresponding enzymes” [6], which contains both traditional and novel types. Nanomaterials with inherent enzymatic catalytic activity have become an attractive research field due to their potential applications. Nanozymes have been broadly utilized in biosensing [7, 8], environmental monitoring and control [9, 10], disease diagnosis and therapy [11, 12], and cytoprotection against harmful biomolecules in cells [13].

**FIGURE 1:**
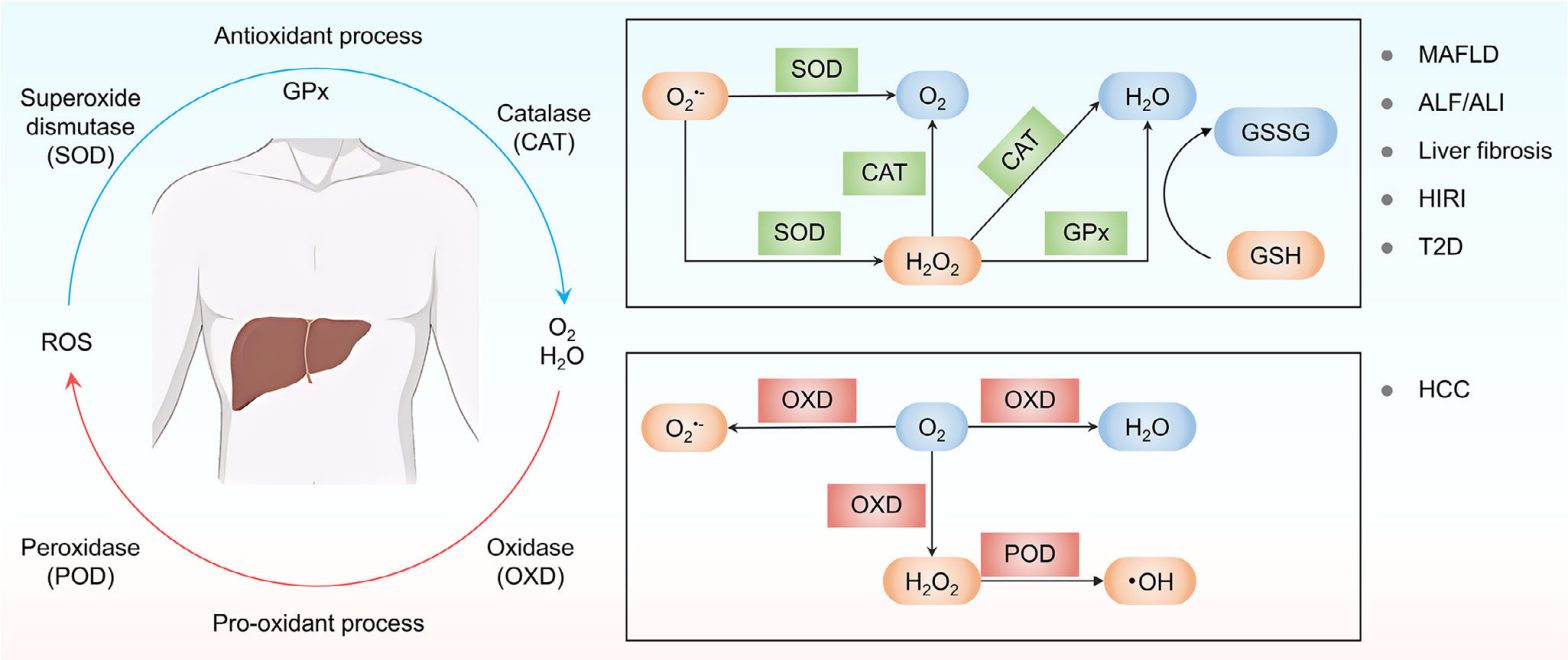
Schematic representation of ROS regulation in liver disease treatment. ROS: reactive oxygen species; GPx: glutathione peroxidase; GSH: glutathione; GSSG: glutathione disulfide; MAFLD: metabolic dysfunction‐associated fatty liver disease; ALF/ALI: acute liver failure/acute liver injury; HIRI: hepatic ischemia‐reperfusion injury; T2D: type 2 diabetes; HCC: hepatocellular carcinoma.

The liver is a central organ for metabolism and detoxification, particularly in regulating oxidative stress. Excessive ROS plays a critical role in the onset and progression of liver diseases such as non‐alcoholic fatty liver disease (NAFLD), liver fibrosis, hepatic ischemia‐reperfusion injury (HIRI), and acute liver injury (ALI) [14]. By mimicking natural antioxidant enzymes, superoxide dismutase (SOD), catalase (CAT), and glutathione peroxidase (GPx), nanozymes can effectively remove ROS, alleviate oxidative damage, and reduce inflammation. In contrast, nanozymes possessing POD‐like activity promote ROS generation and are explored for the treatment of hepatocellular carcinoma (HCC) by inducing tumor cell apoptosis (Figure 1).

Traditional drugs have achieved significant progress in the treatment of liver diseases. However, several challenges remain for patients with liver disorders. For example, sorafenib, the first‐line therapy for HCC, is significantly limited by the development of drug resistance. Resmetirom, the first approved drug for metabolic dysfunction‐associated steatohepatitis (MASH) treatment [15], achieved a 29.9% MASH resolution rate and a 25.9% improvement in fibrosis, compared with 9.7% and 14.2% in the placebo group, respectively [16]. These response rates remain suboptimal and require further improvement. Compared with conventional pharmacological therapies, nanozymes exhibit several distinct advantages in the treatment of liver diseases. Their prolonged circulation time and adjustable delivery vector properties (such as size, morphology, and composition) enhance their liver accumulation. In addition, surface functionalization enables receptor‐mediated delivery to specific liver cell types, including hepatocytes, Kupffer cells (KCs), and hepatic stellate cells (HSCs), facilitating precise therapeutic effects. Despite these promising advancements, several challenges remain. The catalytic efficiency of nanozymes in complex biological environments is still suboptimal, and their targeting precision, biocompatibility, and controllability require further improvement. Moreover, the therapeutic performance of nanozymes is tightly linked to the dynamic liver microenvironment, which is influenced by factors such as pH and temperature.

This review summarizes recent progress in the application of nanozymes for liver disease treatment, focusing on their catalytic mechanisms, delivery strategies, and disease‐specific microenvironments remodeling. We further discuss design principles to enhance their therapeutic performance, explore new approaches such as machine learning, and outline key issues that must be addressed to promote clinical translation.

## 2 Catalytic Mechanism of Nanozymes

**FIGURE 2:**
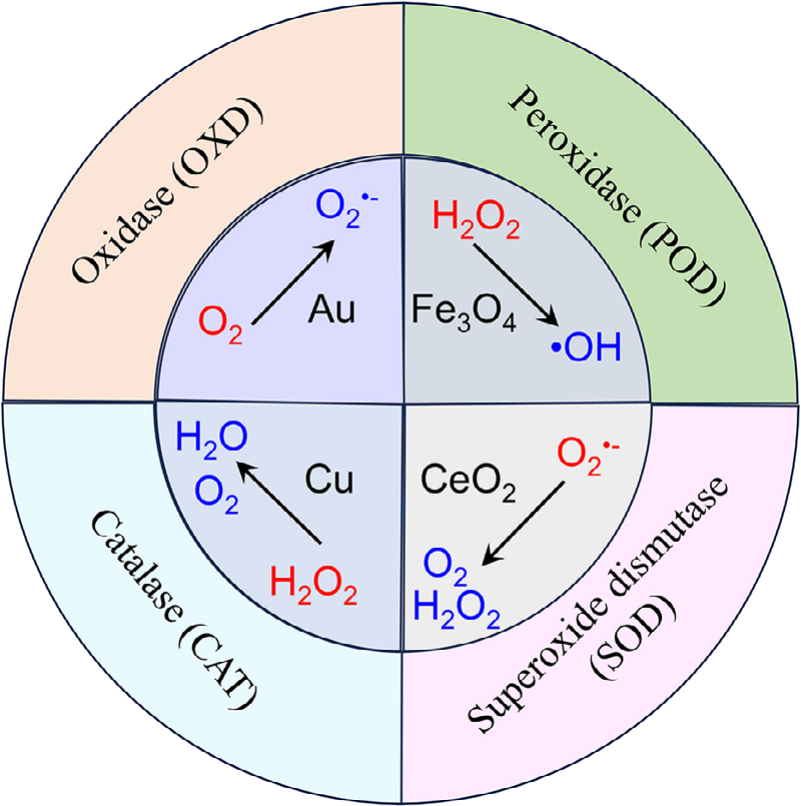
Schematic representation of oxidoreductase‐like nanozymes.

**FIGURE 3:**
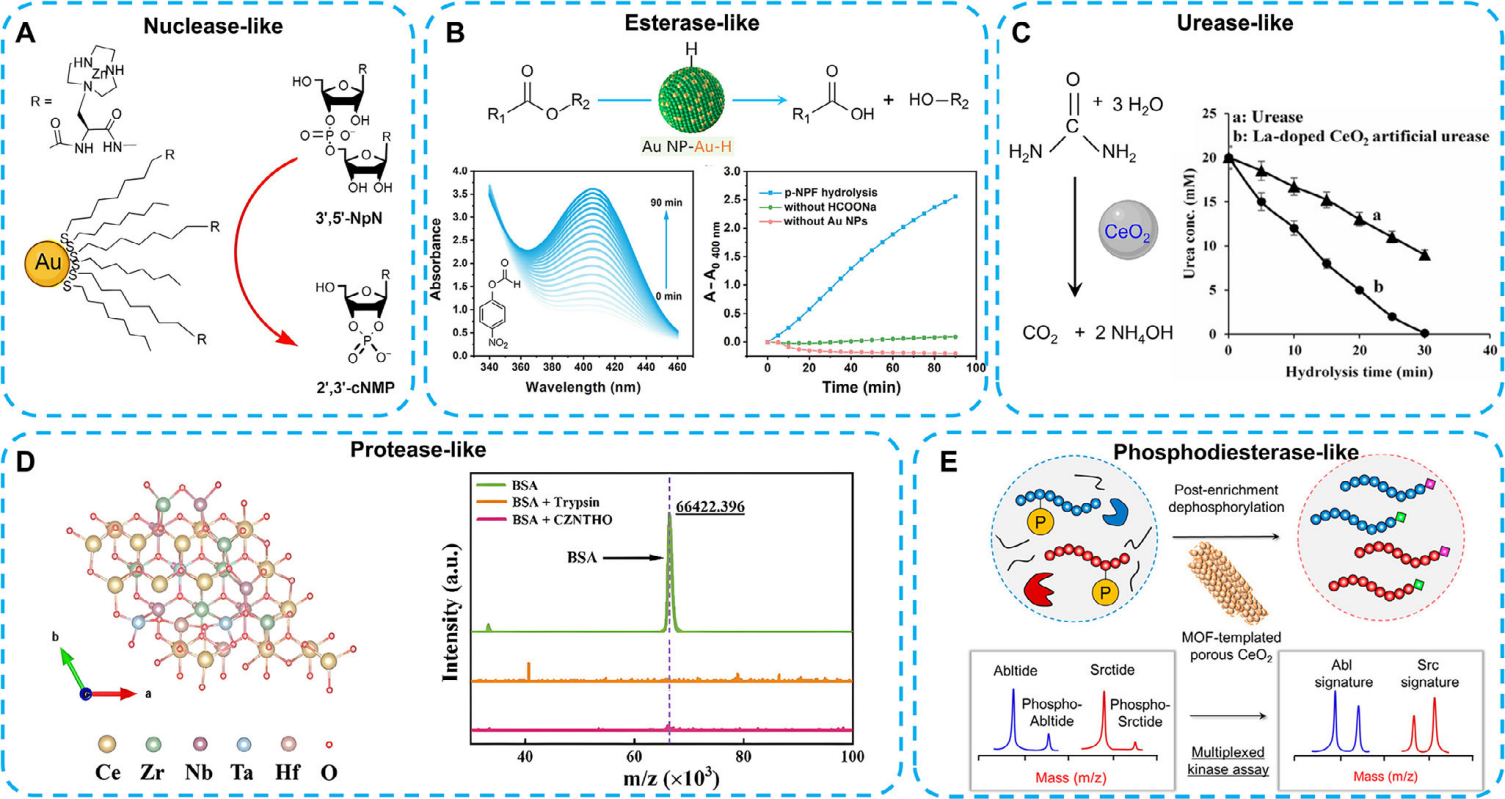
Schematic representation of hydrolase‐like nanozymes with different substrates. (A) Schematic illustration of an Au nanoparticle exhibiting nuclease‐like catalytic activity. Reproduced with permission [2]. Copyright 2004 WILEY‐VCH Verlag GmbH & Co. KGaA, Weinheim. (B) Schematic illustration of an Au nanoparticle exhibiting esterase‐like catalytic activity. Reproduced with permission [30]. Copyright 2024 American Chemical Society. (C) Schematic illustration of La‐doped CeO_2_ nanoparticles functioning as urease‐like nanozymes. Reproduced with permission [33]. Copyright 2025 Elsevier Inc. (D) Schematic of CZNTHO nanozymes exhibiting protease‐like activity. Reproduced with permission [32]. Copyright 2025 Wiley‐VCH GmbH. (E) Schematic of CeO_2_‐based MOFs with PDE‐like catalytic performance. Reproduced with permission [35]. Copyright 2018 American Chemical Society.

To date, numerous nanoparticles with catalytic properties have been identified (Box S1). Based on their catalytic mechanisms, nanozymes can be divided into two categories: oxidoreductases (Figure 2) and hydrolases (Figure 3). Previous studies have demonstrated that most nanozymes exhibit oxidoreductase‐like activity, whereas only a limited number possess hydrolase‐like or other enzyme‐mimicking functions [17]. In this section, we specifically focus on oxidoreductases because of their key roles in regulating ROS in liver‐associated diseases. Moreover, most metabolism‐related enzymatic reactions occur within hepatocytes. Understanding the catalytic mechanisms of hydrolases is critical for elucidating lipid, glucose, purine, and other nutrient metabolic pathways.

Oxidase (OXD) is well‐known for its important role in the evolution of aerobic organisms by effectively utilizing O_2_ as an oxidant to generate O_2_^•−^ or H_2_O_2_. Noble metals (Au), cerium‐based oxides, and manganese‐based oxides have been demonstrated to exhibit OXD‐like catalytic properties [18, 19, 20]. Based on their substrate specificity, OXD nanozymes are further classified as glucose oxidase (GOx)‐like, polyphenol oxidase‐like, cytochrome c oxidase‐like, and laccase‐like nanozymes [21]. POD is a representative enzyme catalyzing substrate oxidation by consuming H_2_O_2_ to produce •OH. Natural PODs can generate high‐valence state intermediates, obtaining electrons from different substrates. Consequently, many nanomaterials exhibit POD‐like properties, including iron‐based magnetic NPs (Fe_3_O_4_), metal oxide NPs (Co_3_O_4_, CeO_2_), carbon‐based NPs (Graphene Oxide, GO), and nanocomposites (GO‐Fe_3_O_4_, Cu‐Ag/rGO, etc.). Recent work has indicated that Fe_3_O_4_ nanozymes exhibit outstanding POD‐like activity through internal electron transfer and the migration of excess Fe ions [22]. CAT is a key enzyme in redox reactions, known for the decomposition of H_2_O_2_ into O_2_ and H_2_O. Many commonly applied nanomaterials have been proven to possess CAT‐like catalytic activities, including metals, metal oxides, and Prussian blue (PB) [23]. Recently, CAT‐like nanozymes have attracted increasing attention for their potential in photodynamic cancer therapy. SOD is a metalloenzyme that catalyzes the disproportionation of O_2_^•−^ to produce H_2_O_2_ and O_2_. SOD nanozymes can be classified as Mn‐SOD, Fe‐SOD, Ni‐SOD, and CuZn‐SOD, based on the different metal cofactors [24]. CeO_2_ nanoparticles are widely regarded as promising candidates for SOD‐like nanozymes. Their outstanding catalytic performance is largely attributed to the Ce^3+^/Ce^4+^ redox states [25]. The formation of oxygen vacancies on the (111) surface of CeO_2_ facilitates oxygen migration and the regeneration of active catalytic sites. During catalysis, the reduction of Ce^4+^ to Ce^3+^ leads to the formation of oxygen vacancies. O_2_ from the surrounding environment is activated by these oxygen vacancies, which enables the reoxidation from Ce^3+^ to Ce^4+^. The dynamic loop of oxygen vacancies endows the effective catalytic performance of CeO_2_ nanozymes [26]. SOD‐like nanozymes have been widely utilized to eliminate the ROS produced during cell damage. Therefore, SOD‐like nanozymes are promising candidates for the treatment of oxidative stress‐related diseases.

Hydrolases are a distinct class of enzymes that catalyze the hydrolysis of chemical bonds, with water acting as the nucleophile in the transfer of functional groups. Based on their hydrolytic substrates, hydrolase‐like nanozymes can be classified as phosphatase‐like, nuclease‐like, protease‐like, esterase‐like, and urease‐like nanozymes [27, 28]. Due to their capacity to degrade macromolecules in biological systems, hydrolase‐like nanozymes play a crucial role in various biological processes. Fullerene‐based nanozymes demonstrated the catalytic ability on the cleavage of the phosphodiester bond of DNA upon irradiation. Au nanozymes act as nuclease‐like [29] and esterase‐like [30] hydrolase nanozymes [31]. Ce_0.5_Zr_0.2_Nb_0.15_Ta_0.1_Hf_0.05_O_x_ (CZNTHO), a high‐entropy metal oxide, exhibits protease‐like activity in hydrolyzing bovine serum albumin (BSA) [32]. La‐doped CeO_2_ functions as a urease‐like nanozyme with promising potential in urea detection and blood cleaning. Compared with natural urease, the cleaning efficiency of La‐doped CeO_2_ was enhanced by 1.8‐fold [33]. Zr‐based MOFs and CeO_2_‐based MOFs both exhibit phosphodiesterase (PDE)‐like activity in cleaving phosphate ester bonds in substrates such as cyclic guanosine monophosphate (cGMP) or cyclic adenosine monophosphate (cAMP) [34, 35].

## 3 Liver Represents a Favorable Organ for Nanozyme‐Based Therapy

### 3.1 The Accumulation of Nanozymes Based on Liver Physiological Characteristics

**FIGURE 4:**
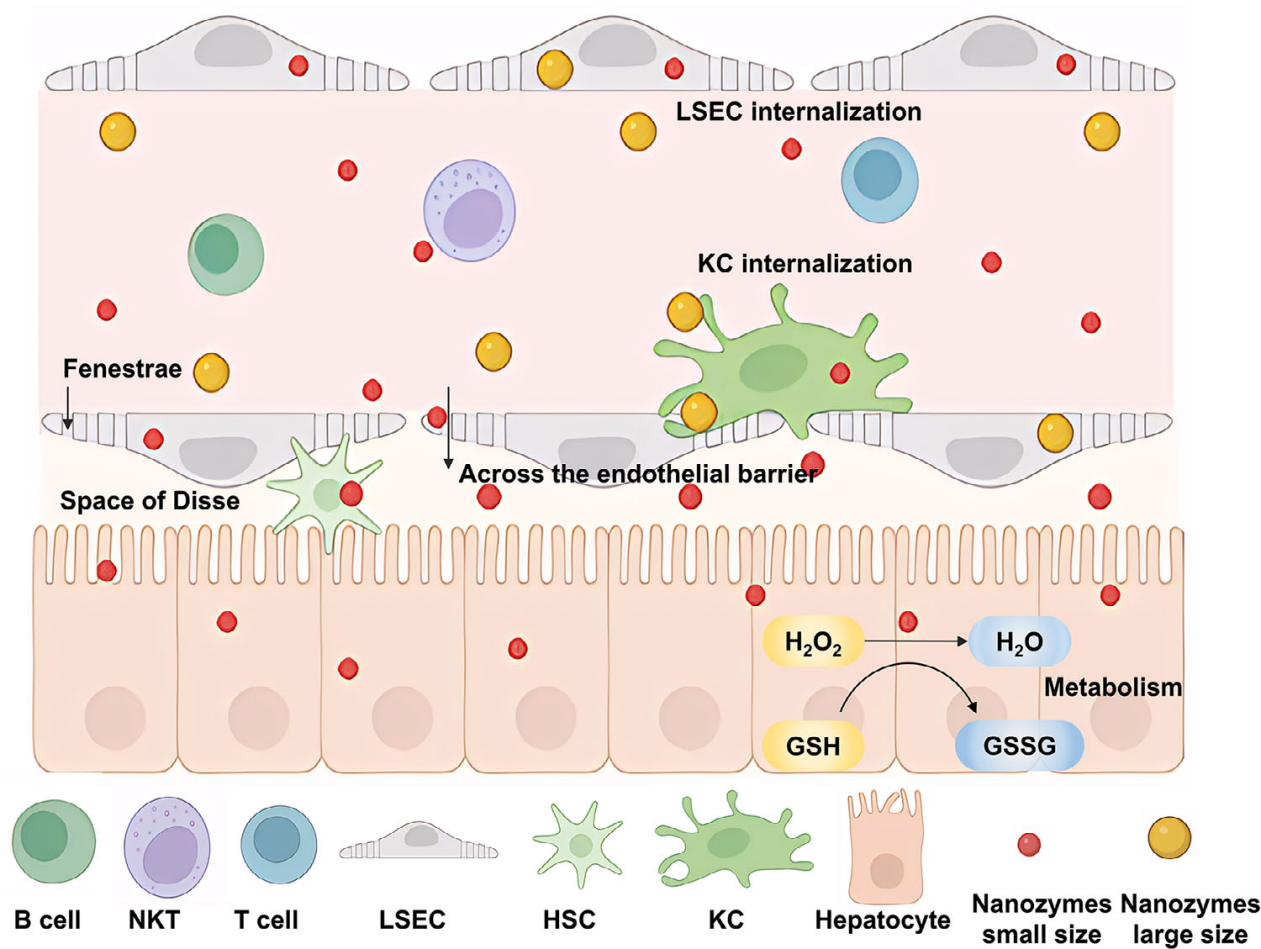
Unique anatomical and circulatory characteristics of the liver relevant to nanozyme‐associated therapies. KC: Kupffer cell; LSEC: liver sinusoidal endothelial cell; HSC: hepatic stellate cell; NKT: natural killer T cell. Schematic created with BioRender.com.

The liver possesses unique anatomical and circulatory characteristics that facilitate the efficient accumulation of nanozymes (Figure 4). The liver is supplied with sufficient blood. It receives nutrient‐rich venous blood from the portal vein (draining the digestive tract and spleen) and oxygen‐rich arterial blood from the hepatic artery [36, 37]. Therefore, nanoparticles administered via intravenous injection or oral route (following intestinal absorption), both commonly used in clinical practice, tend to accumulate in the liver. Moreover, the blood flow rate in the liver sinusoids is substantially lower, thereby prolonging the residence time of circulating components [38].

KCs are liver‐resident macrophages and represent one of the largest populations of tissue macrophages. These cells are responsible for engulfing and eliminating foreign substances and senescent cells from the bloodstream. KCs serve as a key regulator of liver immune microenvironment homeostasis [39]. As exogenous nanoparticles, nanozymes are naturally captured by KCs. Although this uptake may initially appear counterproductive, it can be strategically leveraged to target inflammatory sites. Notably, during liver disease, KCs become critical therapeutic targets for modulating inflammation and oxidative stress. LSECs contain abundant fenestrae (50–300 nm in diameter) on their surface and uniquely lack an underlying basement membrane [40, 41]. This specialized architecture acts as a molecular sieve, allowing nanoparticles of ≤ 200 nm—consistent with the size range of most synthetic nanozymes—to freely enter the space of Disse, where they can directly interact with hepatocytes and HSCs [42]. Collectively, the hepatic vascular architecture, together with the abundance of KCs, positions the liver as an efficient biological filter and natural reservoir for nanoparticle accumulation, thereby enhancing the therapeutic potential of nanozyme‐based interventions.

The liver is a crucial organ in systemic metabolism [43]. Most metabolic enzymes are synthesized in hepatocytes, where they catalyze various metabolic processes. For example, glutathione (GSH) acts as a natural antioxidant, reducing the accumulation of H_2_O_2_ and lipid peroxides [44]. Moreover, as the primary organ responsible for drug detoxification, the liver produces and stores the majority of the body's GSH [45]. The high level of GSH in the liver sinusoids reduces the affinity between Au NPs and serum proteins, altering their blood retention, targeting, and clearance [46]. Similarly, GSH‐rich hepatocytes enable the targeted release of responsive cargo using stimulus‐responsive silica nanoparticles [47]. The synthesis of GSH is often impaired in various liver diseases. In addition, under pathological conditions, the liver tends to accumulate toxic metabolites, leading to ROS production and triggering oxidative stress [48, 49]. SOD‐like and CAT‐like nanozymes are considered ideal antioxidants, directly eliminating excess ROS and thereby alleviating liver damage [50, 51].

### 3.2 Fluctuations in Hepatic pH During the Progression of Liver Disease

The optimal pH range of most nanozymes is relatively narrow compared to the pH gradient present in physiological or pathological liver environments. The pH of normal liver tissue is slightly alkaline (pH = 7.4). However, a classical acidic environment (pH 6.5–6.8) occurs in liver tumors. Acidosis reprograms tumor energy metabolism. The liver is a key regulator of systemic acid‐base homeostasis through lactate metabolism. Nearly 70% of lactate is metabolized in the liver. Lactic acidosis is the most common form of metabolic acidosis and often results from tissue hypoxia secondary to circulatory failure [52]. Albumin, secreted by hepatocytes, is an abundant circulating protein that acts as a weak acid. Hypoalbuminemia, caused by reduced albumin synthesis in liver diseases (e.g., viral hepatitis or cirrhosis), can contribute to mild metabolic alkalosis. In addition, impaired glucose metabolism in diabetes leads to the hepatic generation of ketoacids, culminating in diabetic ketoacidosis in both type 1 and type 2 diabetes mellitus. Nanozymes that exhibit insufficient activity under liver disease pH conditions may limit their therapeutic effects. Conversely, if nanozymes maintain high catalytic activity under physiological pH, off‐target catalytic reactions may occur in non‐diseased tissues, potentially causing adverse effects.

In conclusion, these unique characteristics render the liver one of the most promising and dynamic frontiers in both basic research and clinical translation of nanozymes. Their catalytic properties perfectly match the physiological needs of the liver. In addition, distinctive liver characteristics, including hypoxia, acidic pH, and ROS, are observed in various pathological states, which can serve as the “switch” for catalytic activities of nanozymes. Such controlled catalytic reactions can greatly enhance therapeutic efficacy while reducing the adverse effects. By fully leveraging these inherent advantages, researchers can design more efficient, safer, and smarter nanozyme‐based therapeutics to cure various liver diseases.

## 4 Overcoming Environmental Constraints on Nanozyme Catalytic Activity in the Liver

**FIGURE 5:**
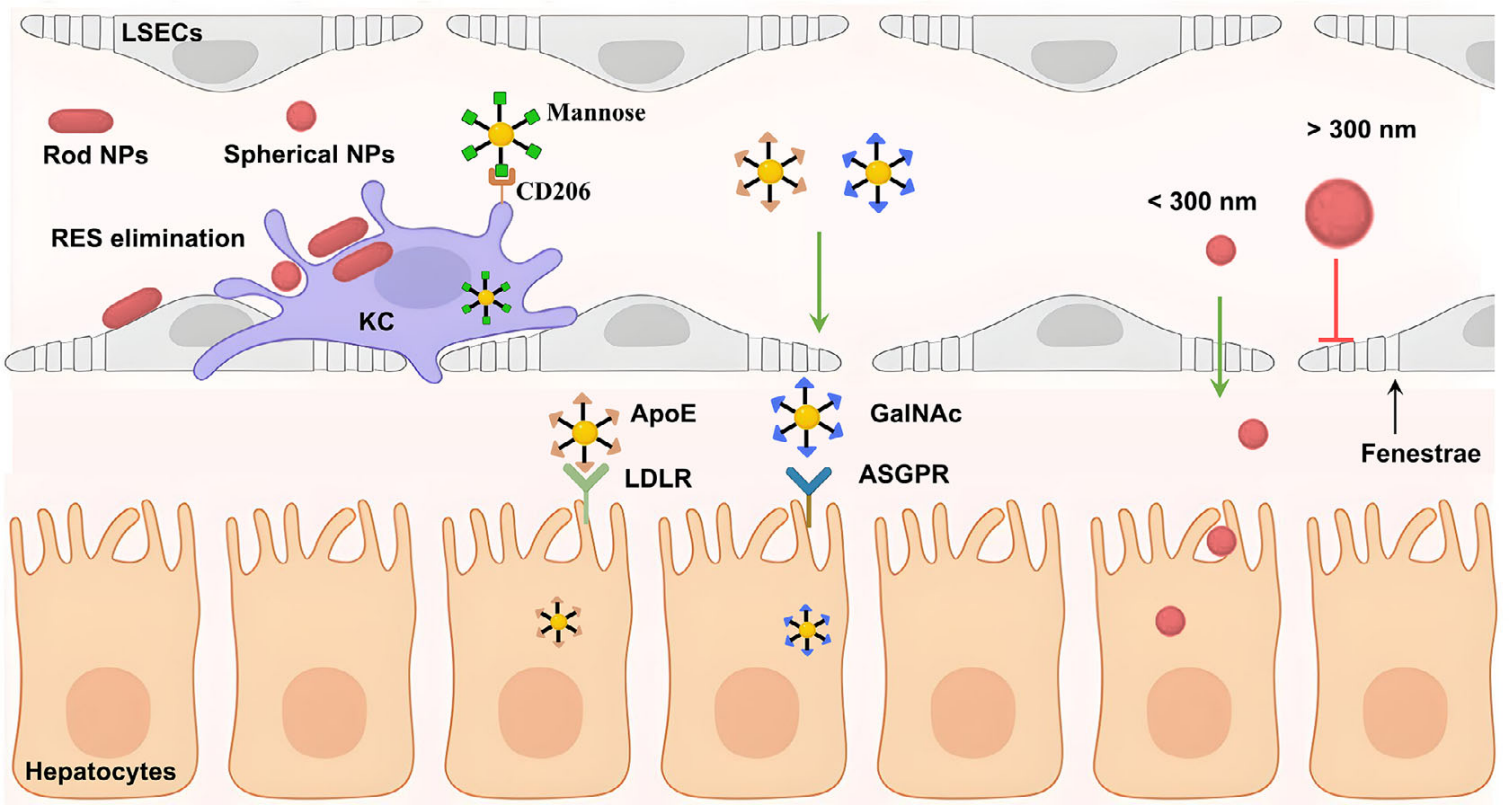
Schematic illustrating representative factors influencing the hepatic delivery efficacy of nanozymes. Created with BioRender.com.

Nanozymes are characterized by various compositions and multiple functions. Owing to their nanoscale architectures, key parameters—including surface area‐to‐volume ratio, substrate accessibility, and the number and distribution of active sites—vary substantially with nanozyme size, morphology, surface modification, and chemical composition (Box S2). To replace natural enzymes, both the activity and selectivity of nanozymes should be optimized. In this section, we first discuss the primary factors in regulating the hepatic delivery efficacy of nanozymes (Figure 5). More importantly, currently available strategies are presented to enable nanozymes to overcome limiting factors under liver‐specific pathological conditions. Optimal catalytic activity of nanozymes is typically achieved under specific pH and temperature conditions, which are rarely encountered in the liver. The precise design of nanozymes within the liver‐specific physiological range improves their catalytic performance. It is well established that the surrounding pH and temperature influence the catalytic performance of nanozymes [53, 54]. For example, Au NPs exhibit POD‐like catalytic activity under acidic conditions, whereas CAT‐ or SOD‐like activities dominate under neutral or alkaline conditions [31, 55]. Therefore, understanding the physiological and pathological characteristics of the liver will greatly benefit the development of nanozyme‐based therapies for liver diseases.

### 4.1 Natural Barriers in the Healthy Liver Sinusoidal Microenvironment

#### 4.1.1 Size Regulation of Hepatocyte Delivery

The majority of nanozymes administered orally or intravenously accumulate in the liver sinusoids via the systemic circulation. Because of the presence of the liver sinusoidal barrier, composed primarily of KCs and LSECs, only a small fraction of nanozymes can reach hepatocytes. The fenestrae on the LSECs range from 50 to 300 nm. Therefore, the size of nanozymes plays a critical role in their delivery to hepatocytes. Although small nanozymes often exhibit superior catalytic properties, they may still be unsuitable for the treatment of liver diseases. In the kidney, the intercellular spacing between glomerular endothelial cells (GECs) is approximately 6 nm. Therefore, systemically administered nanoparticles smaller than 5.5 nm can rapidly traverse the GEC barrier and be eliminated via renal excretion [56]. Nanozymes targeting hepatocytes and HSCs with sizes ranging from 6 to 200 nm may therefore be most effective. However, capillarization, defined as the loss of LSEC fenestrae, occurs during chronic liver inflammation [57], thereby eliminating key trans‐sinusoidal transport pathways.

#### 4.1.2 Morphological Regulation Reduces RES‐Mediated Elimination

In the presence of the hepatic reticuloendothelial system (RES), the majority of nanozymes retained within liver sinusoids are eliminated by KCs and LSECs. To date, the role of nanozyme morphology in liver‐targeted delivery remains poorly understood. Nanozymes with higher aspect ratios tend to remain within blood vessels longer than spherical counterparts, resulting in increased contact with LSECs. Meanwhile, spherical nanoparticles exhibit higher cellular internalization than rod‐shaped counterparts in non‐phagocytic cells. Therefore, when designing nanozymes for hepatocyte‐targeted delivery while minimizing uptake by phagocytic cells such as KCs and LSECs, their morphological characteristics must be carefully considered.

#### 4.1.3 Surface Modification Enables Targeting Delivery

Surface modification plays a vital role in nanoparticle‐based targeting strategies. The clinically approved hepatocyte‐targeting lipid nanoparticle formulation Onpattro relies on apolipoprotein E (apoE) binding‐induced, low‐density lipoprotein receptor (LDLR)‐mediated internalization [58, 59]. In addition, the mannose receptor (CD206) serves as an effective target for KC targeting using mannose‐modified nanoparticles, whereas the asialoglycoprotein receptor (ASGPR) enables hepatocyte‐specific targeting through N‐acetylgalactosamine (GalNAc) modification [60, 61]. Through these surface modification strategies, nanozymes can be selectively delivered to dysfunctional cells, thereby enhancing therapeutic efficacy while substantially reducing the required dose and off‐target adverse effects.

### 4.2 Strategies for Overcoming pH Restrictions on Nanozymes

It is generally known that the POD‐like and OXD‐like activities of nanozymes are favored under acidic conditions, whereas the CAT‐like activity is typically exhibited in neutral or basic environments. To achieve superior catalytic performance of nanozymes for liver disease treatment, strategies are required to overcome pH‐related restrictions (Table 1).

**TABLE 1:** Strategies for overcoming pH restrictions on nanozymes.

Nanozymes	Catalytic activity	pH overcoming mechanism	Reference
Pt	POD	Glucose degradation by GOx	[62]
Pd‐Ru	POD	Glucose degradation by GOx	[63]
Au‐*α*‐FeOOH	POD	Glucose degradation by GOx	[64]
β‐ FeOOH@Fe‐Serine@Au	POD	Glucose degradation by GOx	[65]
Fmoc‐FF	OXD or POD	Diverse carriers provided H^+^	[66]
PCN‐222‐Fe@PAA	POD	Polyacids modification	[67]
Zr‐MOF@PEI/PEGDR	Hydrolase	Polybases modification	[67]
Fe_3_O_4_	POD	Diverse carriers provided H^+^	[68]
Pt	POD	Heparin modification	[69]
CuS‐asp	POD	Aspartic acid modification	[70]
Fe_3_O_4_	POD	ATP modification	[71]
CeO_2_	OXD	ATP modification	[72]
Prussian blue	POD	ATP modification	[73]
Au	CAT	PAMAM‐NH_2_ modification	[74]
2‐line ferrihydrite	CAT	Hydroxyl groups modification	[75]

POD: peroxidase; OXD: oxidase; CAT: catalase; GOx: glucose oxidase; ATP: adenosine triphosphate; PAMAM: polyamidoamine dendrimer.

#### 4.2.1 Nanozymes Directly Remodel the Environmental pH Value

**FIGURE 6:**
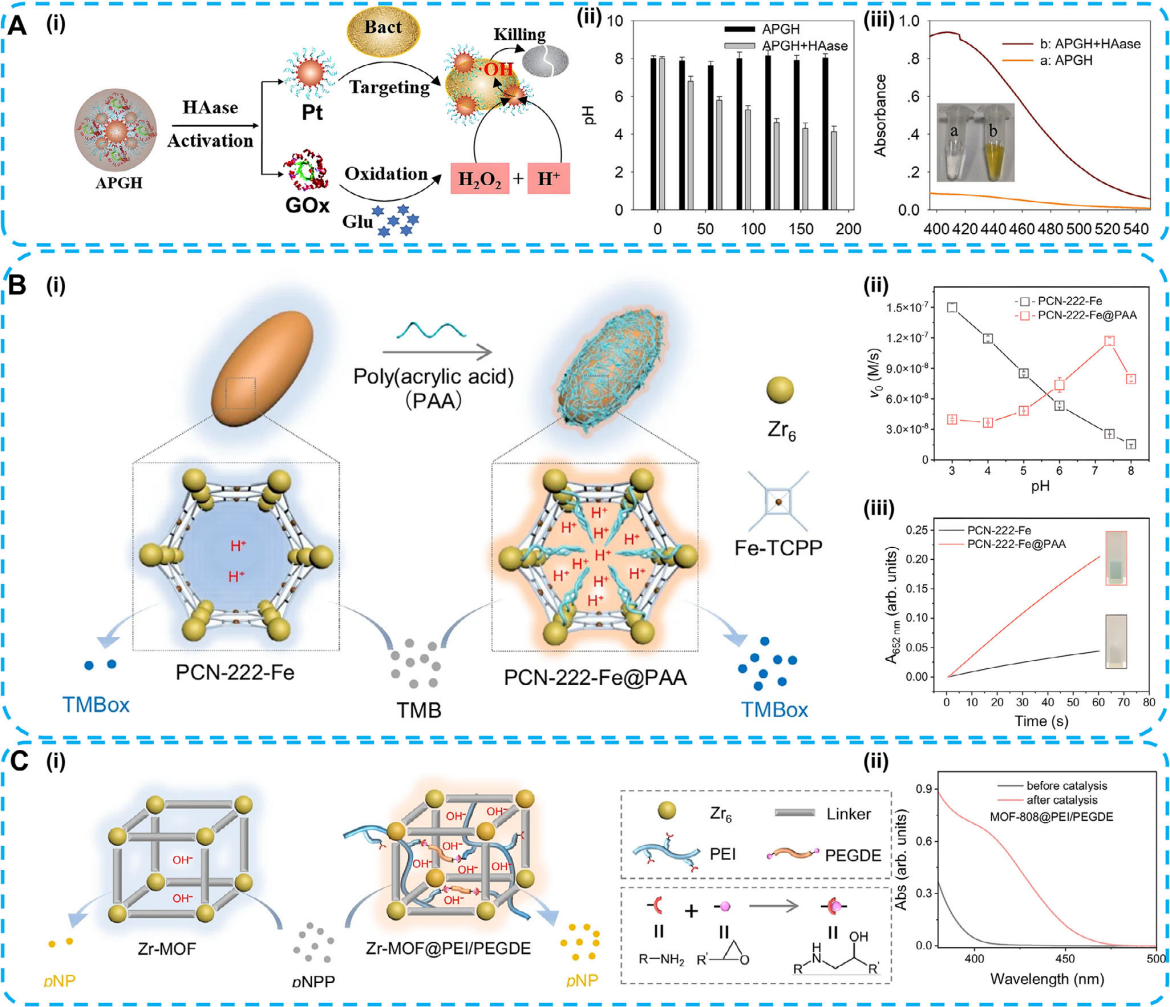
A (i), Schematic illustration of APGH activation, the oxidation of glucose by GOx increased the proton level for decreasing the environmental pH. (ii) and (iii), APGH activation‐induced pH change (ii) and H_2_O_2_ generation (iii) in the PBS buffer (pH 8.0) containing 10 mm Glucose. Reproduced with permission [62]. Copyright 2021 The Author(s). Angewandte Chemie International Edition in English, published by Wiley‐VCH GmbH. B (i), Schematic illustration showing that PCN‐222‐Fe@PAA NPs increase proton concentrations near catalytic sites, resulting in enhanced MOF nanozyme activity. (ii) and (iii), Enhanced peroxidase‐mimicking catalytic activity of PCN‐222‐Fe@PAA NPs at neutral pH. (C) Schematic illustration of microenvironmental pH increase of Zr‐MOFs through poly(ethylene imine) confinement for hydrolytic activity improvement. B and C were reproduced with permission [67]. Copyright 2024 The Author(s). Nature Communications is published by Springer Nature.

A direct and effective strategy to overcome pH restrictions is to adjust the environmental pH to an optimal value. Owing to the abundance of glucose in vivo, an acidic microenvironment can be generated using GOx and Pt nanozyme‐based POD nanocapsules (Figure 6A) [62]. Pd‐Ru nanosheets were combined with GOx to enhance their POD‐like catalytic activity at neutral pH. In the catalytic system, the pH gradually decreased from 7.4 to 3.85, with the addition of glucose [63]. Au NPs also exhibit efficient GOx‐like activity. Upon incorporation of *α*‐FeOOH or β‐FeOOH, Au‐*α*‐FeOOH and β‐FeOOH@Fe‐Serine@Au demonstrate enhanced POD‐like performance under the acidic microenvironment generated by Au NPs [64, 65].

Beyond GOx incorporation, protons required to generate an acidic microenvironment are supplied by diverse carriers. 9‐fluorenylmethoxycarbonyl‐modified diphenylalanine (Fmoc‐FF) hydrogel releases protons at neutral pH through dissociation of the carrier. The Pt nanozymes loaded in the hydrogel exhibit OXD‐ or POD‐like catalytic activity at neutral or even alkaline pH conditions [66]. Polyacids or polybases incorporated into the MOF nanozymes regulate the microenvironmental pH level. Poly(acrylic acid) (PAA) (as Brønsted acids) confined within the channels of Zr‐MOF (PCN‐222‐Fe NPs) lowers the local microenvironmental pH, thereby enhancing POD‐like catalytic activity (Figure 6B). In addition, poly(ethylene imine) (PEI), acting as a Brønsted base and incorporated into Zr‐MOF nanozymes, increases the local microenvironmental pH, thereby enabling hydrolase‐like activity (Figure 6C) [67]. Similarly, Fe_3_O_4_ nanozymes loaded into the aluminium oxide nanochannel vector exhibit outstanding POD‐like activity at pH 7.0. This spatial confinement within aluminium oxide nanochannels facilitates protonation, thereby decreasing the in‐pore pH to approximately 5.0 [68].

#### 4.2.2 Enhancing Nanozyme Catalytic Performance under Physiological Conditions

**FIGURE 7:**
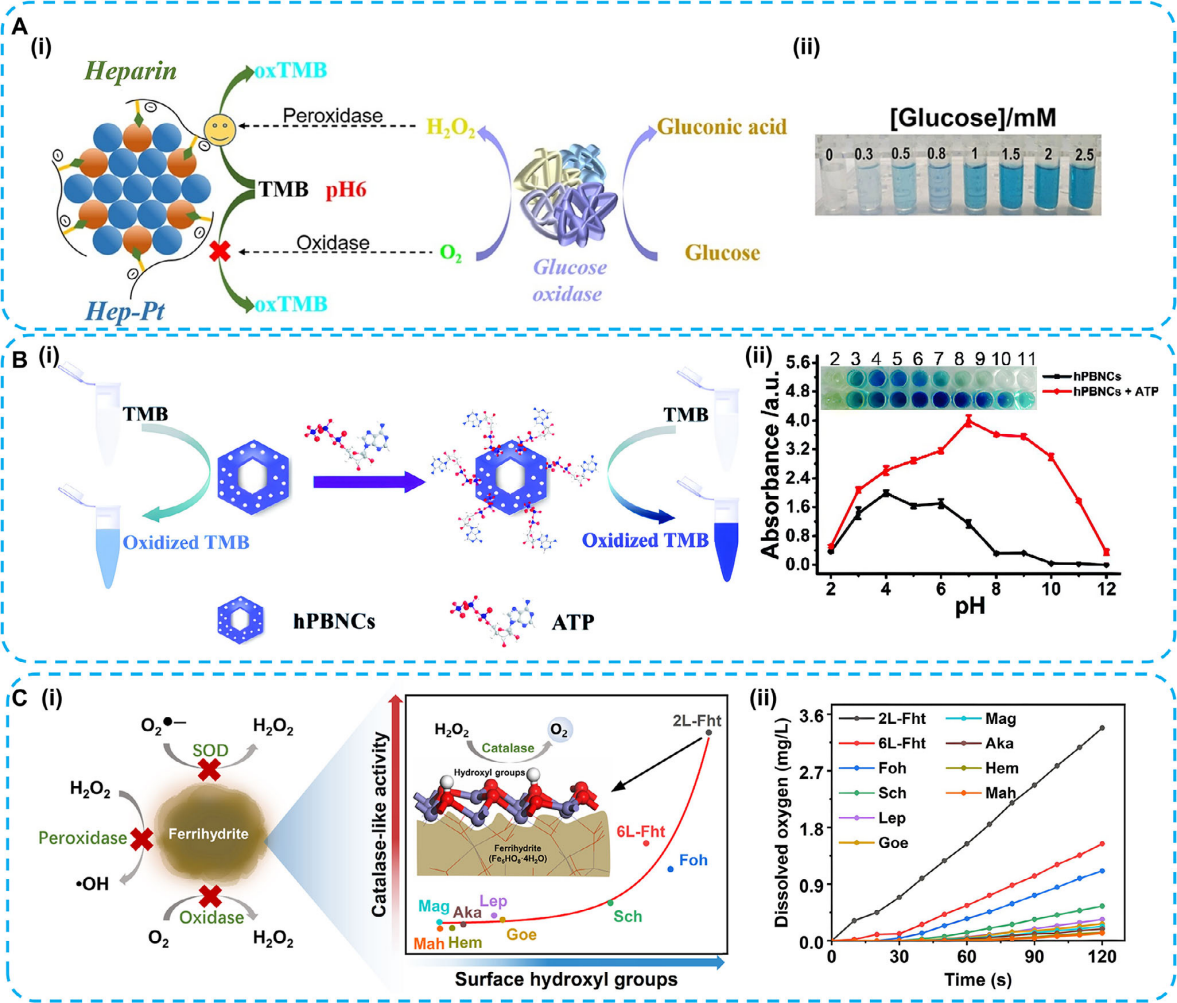
(A). Schematic diagram for the application of heparin‐modified Pt nanozymes with sufficient POD‐like activity at pH 6 in glucose detection. Reproduced with permission [69]. Copyright 2020 The Author(s). Elsevier Ltd. (B) Schematic illustration of the ATP‐induced enhancement of the peroxidase‐like activity of hPBNCs at a broad range (pH 2–12). Reproduced with permission [73]. Copyright 2020 The Royal Society of Chemistry. (C) Schematic illustration of ferrihydrites’ catalase mimicking activity enhanced with surface hydroxyl groups addition. Reproduced with permission [75]. Copyright 2021 The Author(s). Published by Elsevier Ltd.

Another strategy to enhance the therapeutic efficacy of nanozymes under non‐optimal pH conditions is to directly improve their catalytic activity. This can be achieved by modulating the interactions between nanozymes and their substrates. Certain surface modifications can promote electrostatic adsorption of substrates. Heparin‐modified Pt nanozymes increase the surface negative charge, thereby enhancing the adsorption of positively charged substrates (3,3',5,5'‐Tetramethylbenzidine, TMB). At pH 6.0, the POD‐like catalytic activity remained 60% of maximum (Figure 7A) [69]. Aspartic acid (asp, isoelectric point: 2.97) modification provides abundant negative charges under neutral and alkaline conditions. CuS‐asp nanozymes displayed POD‐like catalytic activity at neutral pH [70].

ATP is a commonly employed strategy to overcome the pH limitations of nanozymes. The POD‐like catalytic property of Fe_3_O_4_ nanozyme was significantly enhanced under neutral pH conditions through enhanced adsorption and stabilization of the TMB substrate by ATP [71]. In addition, phosphate anions released from ATP at pH 7.4 can generate O_2_^•−^, thereby enhancing the OXD‐like properties of CeO_2_ nanozymes [72]. Hollow Prussian blue nanocubes (hPBNCs) offer abundant binding sites for ATP due to their inherent porous structures. The optimal POD‐like catalytic activity is adjusted from pH 4 to 7. Moreover, ATP addition amplifies the catalytic activity of hPBNCs over a broad pH range (2–12) (Figure 7B) [73].

Functional group modification effectively tunes the catalytic activity of nanozymes. The amine group at the terminus of polyamidoamine dendrimer (PAMAM) modified on Au nanozymes is easily protonated under the acidic tumor microenvironment (TME), thereby enhancing the adsorption of OH^−^. Au‐PAMAM‐NH_2_ nanozymes exhibit optimal CAT‐like activity over the physiological pH range (pH 4.8 to 7.4), further enhancing photodynamic therapy efficacy to overcome cancer cell hypoxia [74]. The hydroxyl groups on iron oxide nanomaterials are critical determinants of CAT‐like catalytic activity. 2‐line ferrihydrite, with abundant surface iron‐associated hydroxyl groups, exhibits the highest CAT‐like activity in the pH range of 4.0–8.7 (Figure 7C) [75].

### 4.3 Low Temperature Regulatory Switching the Catalytic Activity

Temperature restricts the catalytic performance of both enzymes and nanozymes. Most natural enzymes exhibit catalytic activity under mild conditions, whereas only a few can maintain activity in extreme environments. In contrast, numerous nanozymes sustain biocatalytic activity across a wide temperature range due to their intrinsic stability. Nanozymes, exhibiting robust performance at physiological temperatures (around 37°C), can serve as direct replacements for natural enzymes in vivo.

**FIGURE 8:**
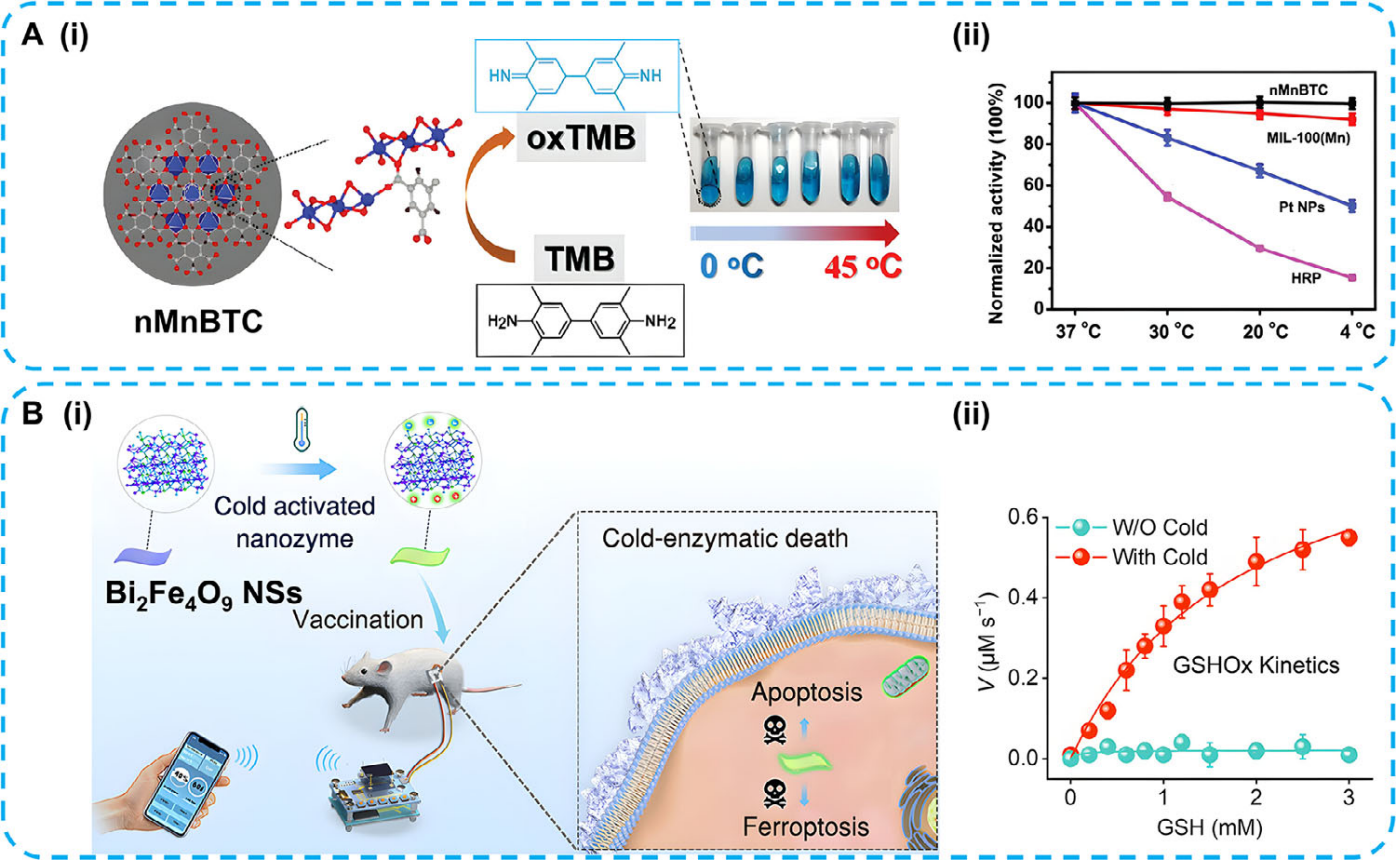
(A) (i), Scheme of nMnBTC synthesis and its catalytic behavior with temperatures ranging from 0 to 45°C. (ii) Comparison of the normalized catalytic activity of nMnBTC, MIL‐100(Mn), Pt NPs, and HRP at varied temperatures. Reproduced with permission [76]. Copyright 2022 Wiley‐VCH GmbH. (B) (i), Schematic diagram of cold‐nanozyme (Bi_2_Fe_4_O_9_ nanosheets)‐mediated catalysis of antitumor immunity. (ii) Enzymatic kinetics of GSH‐OXD‐mimic activity of Bi_2_Fe_4_O_9_ under normal and cold conditions. Reproduced with permission [77]. Copyright 2022 American Chemical Society.

Some nanozymes maintain high catalytic activity at low temperatures. For example, nMnBTC nanozymes exhibit outstanding OXD‐like properties from 0 to 45°C (Figure 8A) [76]. The safe low‐temperature range (4°C–37°C) for the human body is substantially broader than the tolerable high‐temperature range (37°C–42°C). For most natural enzymes, reduced activity and rapid deactivation typically occur under cold conditions. Therefore, cold‐adapted nanozymes attract considerable interest for biomedical applications. The Bi_2_Fe_4_O_9_ nanosheets exhibit robust glutathione oxidase (GSH‐OXD)‐like catalytic activity at low temperatures. The required low‐temperature conditions are enabled by their pyroelectric properties. Bi_2_Fe_4_O_9_ nanosheets enhance antitumor immunity by triggering cold‐activated enzymatic death of tumor cells via apoptosis and ferroptosis. This cold‐activated strategy minimizes off‐target toxicity in normal tissues for in vivo tumor vaccine applications (Figure 8B) [77].

### 4.4 Laser‐ or Light‐Induced Thermal Regulation of the Microenvironment

Laser irradiation is widely used to stimulate the properties of nanomaterials by providing reaction energy and elevating the local temperature. Light is considered an effective tool for inducing changes in pH and temperature. Single‐atom nanozymes (SAzymes) exhibit excellent POD‐ and CAT‐like activities, but their catalytic efficiency is often compromised in the TME. Therefore, iridium (Ir) SAzymes combined with natural GOx enzymes serve as a dual‐enzyme‐driven cascade system for tumor catalytic therapy under laser irradiation. Upon laser irradiation, Ir SAzymes increase the local temperature, thereby enhancing GOx activity and generating abundant H_2_O_2_ to decrease the local pH. By optimizing pH and temperature, the key reaction‐limiting factors, the Ir SAzymes‐GOx dual‐enzyme system significantly promotes tumor catalytic therapy [78].

**FIGURE 9:**
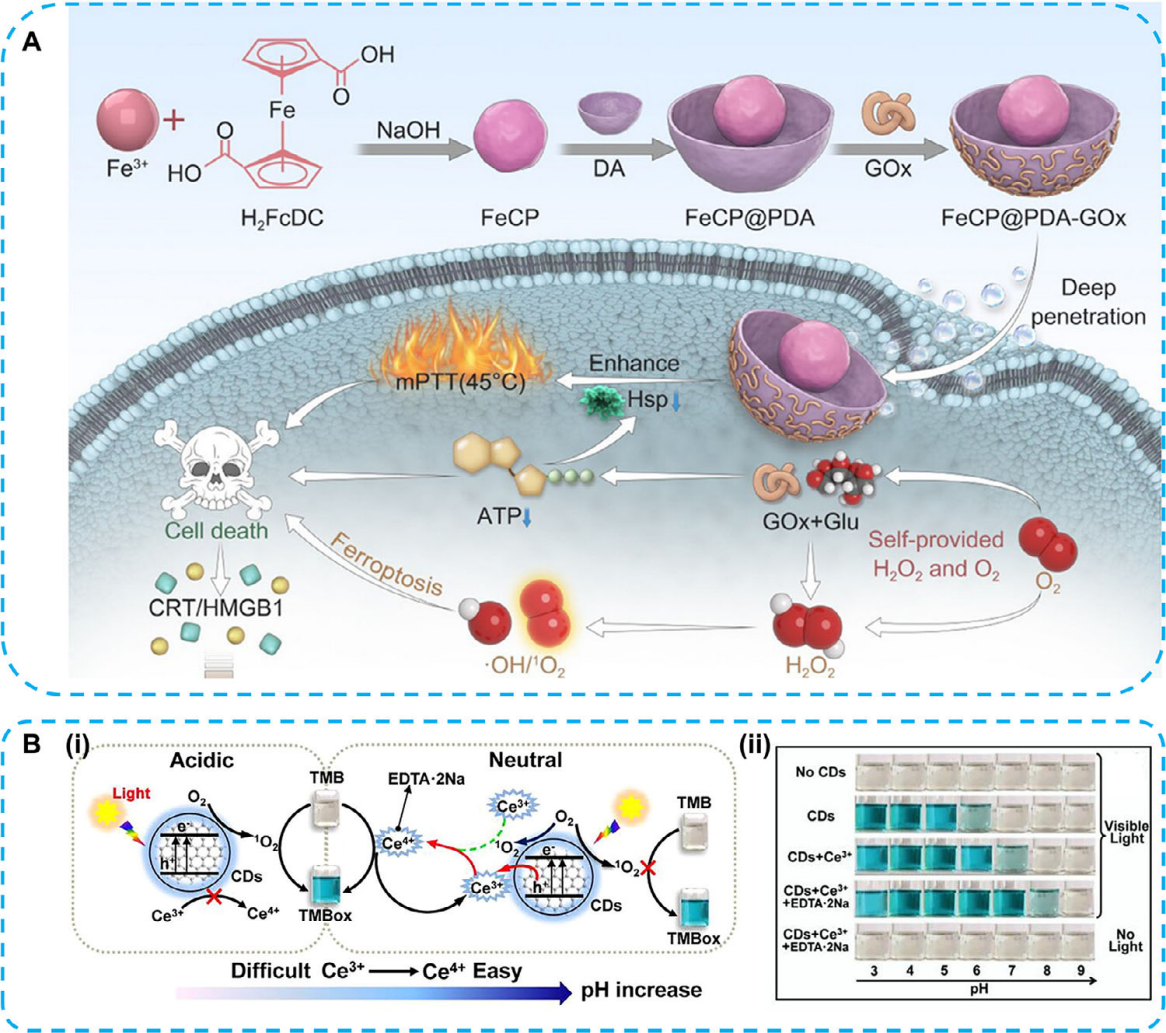
(A) Schematic illustration of the multifunctional nanosystem (FeCP@PDA‐GOx) inducing ferroptosis with mPTT. Reproduced with permission [79]. Copyright 2025 Wiley‐VCH GmbH. B (i), Illustration and (ii) neutral preferred OXD‐like performance of C‐dots (Carbon dots) mediated by Ce^3+^ and EDTA‐2Na under light irradiation. Reproduced with permission [80]. Copyright 2020 Elsevier.

Mild photothermal therapy (mPTT), which maintains tissue temperatures in the range of 42–45°C, is widely used to suppress tumor growth and promote the release of tumor‐associated antigens, thereby activating antitumor immune responses. mPTT alleviates hypoxia within the TME, providing an optimal environment for GOx activity. Mei et al. constructed a multifunctional, multienzyme nanosystem (FeCP@PDA‐GOx) for melanoma therapy. This system compensated for insufficient intratumoral H_2_O_2_ levels through GOx‐mediated catalysis and further amplified the catalytic performance of FeCP@PDA (Figure 9A) [79].

C‐dots and Ce^3+^ comprised nanozymes exhibit light‐responsive catalytic activity at neutral pH. The redox potential of the Ce^3^^+^/Ce^4^^+^ couple varies with pH, allowing the band energy levels of C‐dots to be modulated by changes in pH. Moreover, Ce^4+^ stabilized by ethylenediaminetetraacetic acid disodium salt (EDTA‐2Na) promotes the oxidation of TMB at pH 7.0 (Figure 9B) [80]. Photosensitizer malachite green carbinol base (MGCB) and GO were hybridized to generate OH^−^ from MGCB and high temperature from GO respectively, upon irradiation with ultraviolet and near‐infrared light [81]. A wide range of both pH and temperature can be achieved under light stimulation. Therefore, nanomaterials with photosensitizing properties may further improve catalytic activity for liver disease therapy.

Overall, rational regulation of structural features and external stimuli provides versatile strategies to enhance nanozyme performance. The size, morphology, and surface modification of nanozymes determine their biodistribution in the liver. Regulation of such parameters has promising potential in liver‐targeting delivery at both organ and cellular levels. Adjustments to composition or hybridization, as well as molecular modifications of nanozymes, promote their catalytic performance by overcoming environmental or physiological restrictions. Both low‐ and high‐temperature stimuli are regarded as viable approaches for intelligently controlling catalytic reactions. Future efforts should integrate these parameters with liver‐specific targeting requirements, paving the way for more effective and safe therapeutic applications.

## 5 Applications of Nanozymes in Liver Disease Therapy

Liver disease causes approximately 2 million deaths annually, accounting for 4% of total deaths [82]. Currently, liver disease is the eleventh‐leading cause of death, but liver‐associated deaths may be underestimated [83]. Moreover, cirrhosis often progresses to HCC [84, 85, 86]. It is estimated that 43% of the global population consumes alcohol, and excessive alcohol consumption is a major risk factor for illness, disability, and mortality [87]. With the rising prevalence of obesity, the global prevalence of NAFLD is estimated to be 32.4% and continues to increase [88]. Acute liver failure (ALF) is most commonly caused by drug‐induced liver injury and represents the primary indication for emergency liver transplantation. Alcohol‐, lipid‐, and other metabolic dysfunction‐associated liver diseases are challenged by redox imbalance [82, 89, 90]. Therefore, nanozymes possess broad application potential in liver disease treatment.

### 5.1 Metabolic Dysfunction‐Associated Fatty Liver Disease (MAFLD)

With the rising prevalence of obesity, patients diagnosed with metabolic dysfunction‐associated fatty liver disease (MAFLD) are at an increased risk of developing MASH, previously referred to as nonalcoholic steatohepatitis (NASH). The global prevalence of MAFLD, including steatohepatitis, cirrhosis, and HCC, has increased 20%–40% [91]. Currently, the U.S. Food and Drug Administration (FDA)‐approved pharmacological interventions (resmetirom) for MASH are effective only in patients who have already developed liver fibrosis [15]. Individuals with MAFLD who consume excessive high‐fat diets experience severe lipid and oxidative metabolic stress, resulting in overproduction of ROS, mitochondrial dysfunction, and endoplasmic reticulum stress [92]. The accumulation of ROS and the induction of inflammatory responses further promote the progression of MAFLD to MASH [93]. Nanozymes represent a viable intervention strategy for metabolic dysfunction‐associated liver disorders, offering unique antioxidant and anti‐inflammatory effects. Resmetirom promotes the β‐oxidation of fatty acids in MASH disease. Comparing with clinical drug, many nanozymes exhibit dual therapeutic effects both on hepatocytes and macrophages.

**FIGURE 10:**
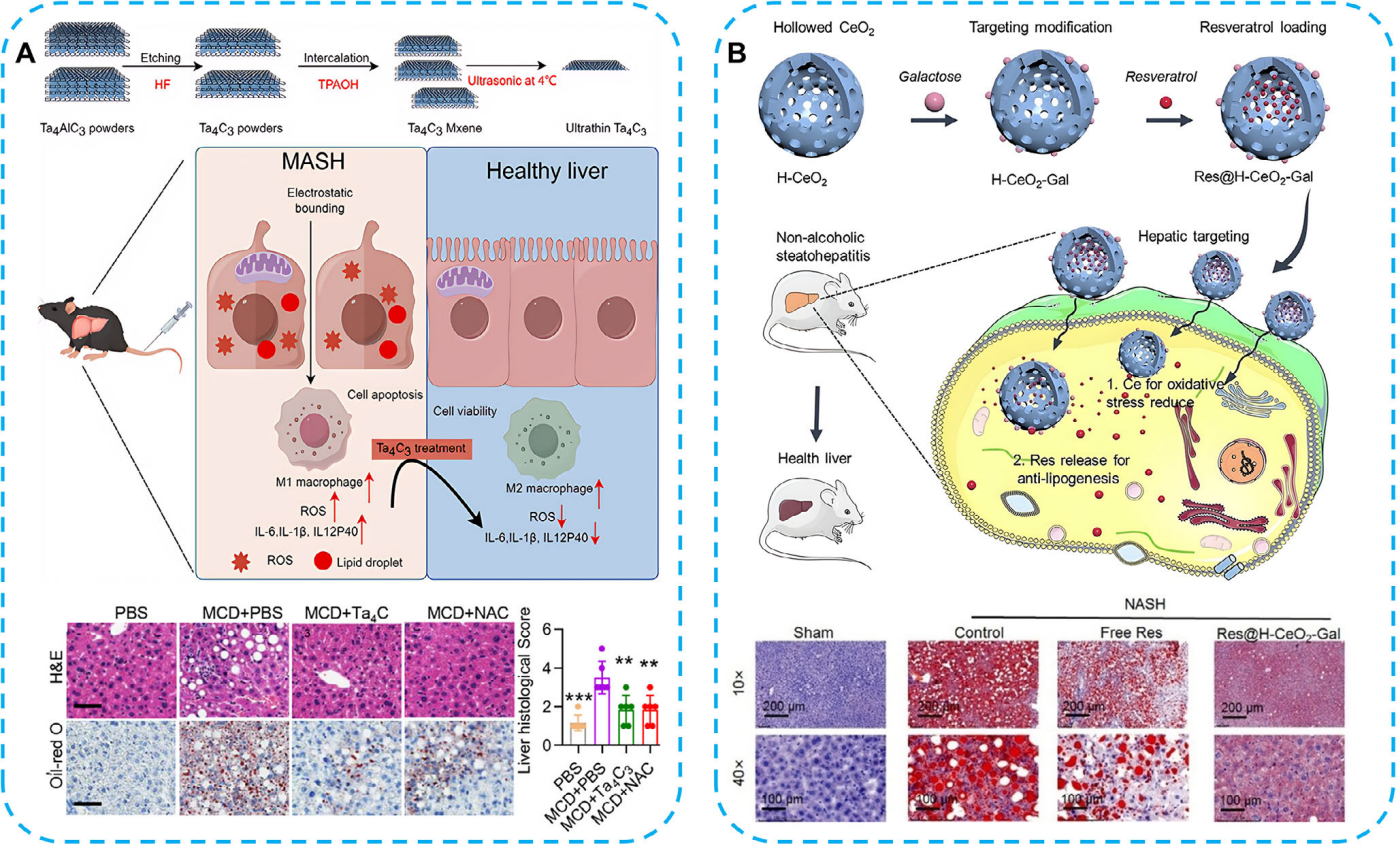
(A) The Ta_4_C_3_ nanozyme exhibited SOD‐like catalytic activity by scavenging ROS and suppressing inflammatory cytokine levels in MASH. Reproduced with permission [95]. Copyright 2025, American Chemical Society. (B) Schematic illustration of Res@H‐CeO_2_‐Gal fabrication for precise liver targeting and resveratrol delivery for MASH treatment via inflammation prevention and anti‐adipogenesis. Reproduced with permission [97]. Copyright 2022 Wiley‐VCH GmbH.

Two‐dimensional (2D) materials have attracted great interest in biological applications. MXenes consist of metal (M) layers interleaved with carbon or nitrogen (X) layers, such as Ti_2_C, Ta_4_C_3_, M_2_X, M_3_X_2_, and M_4_X_3_ [94]. He et al. employed 2D materials Ta_4_C_3_, a MXene‐based nanozyme, for the antioxidant treatment of MAFLD. Following Ta_4_C_3_ injection, lipid droplet accumulation in hepatocytes was significantly inhibited; excessive ROS was eliminated through its catalytic activities; the expression and secretion of tumor necrosis factor‐α (TNF‐α), Interleukin‐6 (IL‐6), and Interleukin‐1β (IL‐1β) were significantly downregulated. This study demonstrated that Ta_4_C_3_ nanozyme possesses multiple bioactive functions, including alleviating inflammation and eliminating ROS generated by hepatocytes and macrophages (Figure 10A) [95]. In another obesity‐induced MAFLD rat model, nanocrystalline cerium dioxide (nCeO_2_) was utilized owing to its excellent SOD‐like and CAT‐like catalytic activities. nCeO_2_ exerted antioxidant effects by scavenging excessive ROS in the liver, preventing the secretion of proinflammatory cytokines (IL‐1β, IL‐12), and promoting the release of anti‐inflammatory cytokines (IL‐4, IL‐10, TGF‐β). nCeO_2_ nanozymes successfully prevented the obesity‐induced liver damage [96].

To further improve the ROS scavenging ability, cationic liposomes were employed for intracellular delivery. Self‐assembling cerium nanoparticles were loaded onto the inorganic calcium phosphate core and combined with cationic liposomes to form Ce‐SANPs. This strategy enhanced the clearance of ROS in hepatocytes, providing an efficient therapy for MAFLD. As MAFLD progresses to MASH, additional pharmacological interventions are required for effective treatment. Bao and colleagues designed a liver‐specific targeting nanozyme delivery system based on galactose modification. A hollow CeO_2_ (H‐CeO_2_) structure was prepared to carry resveratrol (Res), forming Res@H‐CeO_2_‐Gal. Under MASH conditions, hepatocytes experienced lipid and oxidative metabolic stress. The enhanced accumulation of Res@H‐CeO_2_‐Gal in the liver reduced lipid accumulation via Gal modification and promoted anti‐inflammatory responses, depending on the antioxidant capacity of Ce‐based nanozymes. These results indicate a promising liver‐targeted nanozyme delivery system, offering a novel strategy for alleviating MASH and other liver disorders (Figure 10B) [97].

### 5.2 Acute Liver Failure, Liver Fibrosis, and Hepatic Ischemia‐Reperfusion Injury

#### 5.2.1 Acute Liver Failure

The liver is one of the vital organs in the human body, responsible for maintaining fundamental metabolic processes. ALF is a multifactor‐induced fatal liver disease, characterized by rapid hepatocyte dysfunction. Although rare, ALF most frequently occurs in otherwise healthy adults without any preexisting liver disease [98]. ALI can be considered the initial stage of ALF; without prompt and effective intervention, ALI may ultimately progress to ALF and lead to death [99]. ALI has become a global public concern due to its susceptibility to common risk factors such as adverse drug reactions, alcohol abuse, viral infections, autoimmune hepatitis, and injuries from hepatic resection or transplantation [100, 101, 102]. With a growing understanding of the pathophysiological processes of ALI, ROS are now recognized as playing a predominant role in inducing liver necrosis. The major forms of ROS include O_2_^•−^,·OH, and H_2_O_2_ [41]. Nanozymes are emerging nanomaterials with enzyme‐like catalytic characteristics and are expected to potentially replace N‐acetylcysteine (NAC) and GSH for scavenging ROS in the liver.

**FIGURE 11:**
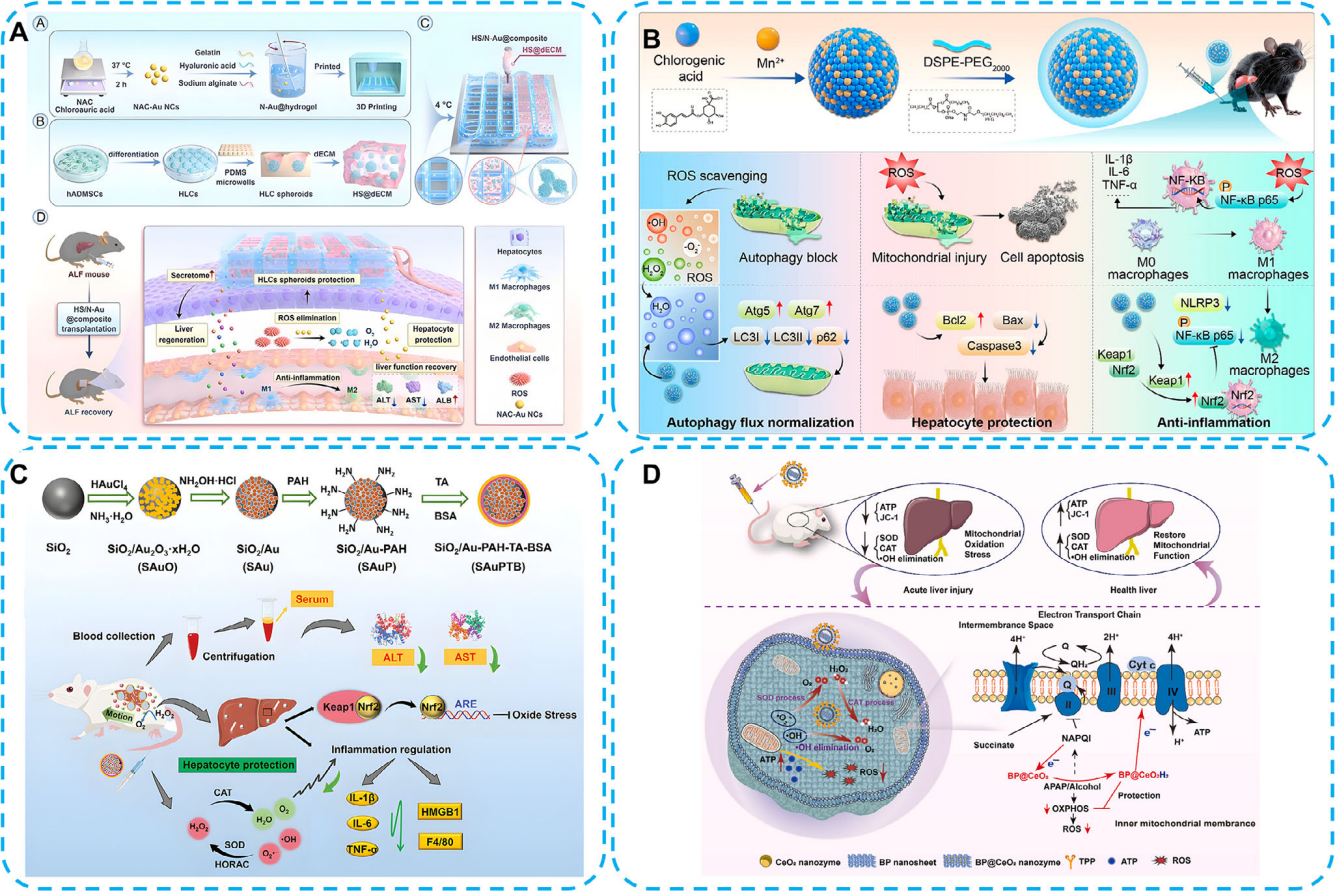
(A) Schematic illustration of the combination of HLC spheroids and NAC–Au NC nanozymes via 3D‐printed composite scaffolds for ALF therapy. Reproduced with permission [104]. Copyright the Author(s) 2024 Elsevier Ltd. (B) Schematic diagram of metal–phenolic nanozymes (CA‐Mn NPs) for the treatment of ALI. Reproduced with permission [106]. Copyright 2024 American Chemical Society. (C) Schematic representation of the synthetic procedures and therapeutic mechanism of SAuPTB. Reproduced with permission [108]. Copyright 2023 Wiley‐VCH GmbH. (D) Schematic illustration of the mechanism of TBP@CeO_2_ nanozymes for treating ALI. Reproduced with permission [110]. Copyright 2025 Elsevier Ltd.

Considering the rapid onset of drug‐induced ALI, combined therapeutic approaches are essential. Stem cell therapy offers a promising solution for hepatocyte necrosis, although its effectiveness is limited by the ROS‐enriched microenvironment [103]. Ultrasmall gold nanoclusters (Au NCs) (< 2 nm) exhibit promising SOD‐ and CAT‐mimicking catalytic activities, thereby enhancing the viability of transplanted cells. NAC, an FDA‐approved antidote for drug‐induced liver injury, is an ideal payload for Au NCs. Jin et al. used NAC‐Au NCs as proliferation stimulators in hepatocyte‐like cells (HLCs) within 3D‐printed composite scaffolds. In CCL_4_‐ or acetaminophen (APAP)‐induced ALF mouse models, NAC‐Au NCs enhanced HLC viability and functions by eliminating ROS and suppressing inflammatory responses. The in situ transplanted HLCs secreted liver regeneration factors, thereby protecting hepatocytes and improving liver function. This novel research ingeniously combined bio‐printed Au nanozymes with stem cell in situ transplantation, effectively utilizing nanomaterials as both delivery vehicles and regulatory agents (Figure 11A) [104].

Ferroptosis, a novel ROS‐dependent form of cell death, has increasingly been recognized in ALI and other inflammatory diseases [105]. To prevent hepatocyte ferroptosis, Xiao's group designed a novel metal‐phenolic nanozyme (CA‐Mn NPs) for an antioxidant therapy. By mimicking SOD‐like catalytic activity, CA‐Mn nanozymes regulated the production of excessive ROS in the CCl_4_‐induced mouse ALI model. Furthermore, they revealed the underlying anti‐inflammatory mechanisms of CA‐Mn nanozymes by regulating the Nrf2‐Keap1 and NF‐κB p65 pathways in macrophages. This work offers a novel strategy for ALI therapy that goes beyond alleviating classical oxidative stress to specifically prevent ferroptosis (Figure 11B) [106]. In addition, natural polyphenols (catechin) exhibited outstanding inhibition of ferroptosis [107], providing a compelling approach for constructing natural product‐nanozyme hybrids for future ALI treatment.

Eliminating ROS is not the sole objective of ALI treatment. Zhou et al. employed SiO_2_ nanoparticles as another ideal carrier for ultrasmall gold nanoclusters. The ultrasmall Au nanoclusters were first loaded onto SiO_2_, then chelated with tannic acid, and finally stabilized with BSA (SAuPTB). This multifunctional nanozyme exhibited outstanding therapeutic effects by scavenging ROS, promoting hepatocyte proliferation, and alleviating the inflammatory response (Figure 11C) [108]. Mitochondria are the primary source of excessive ROS in liver diseases [109]. Therefore, a mitochondrial targeting strategy holds promising therapeutic potential for ROS elimination. Liang et al. designed a two‐pronged black phosphorus/Ceria nanozyme (TBP@CeO_2_) for antioxidant therapy in ALI. In addition to providing SOD‐ and CAT‐like catalytic activities, TBP@CeO_2_ also restored mitochondrial function by recovering complex II activity in the electron transport chain. This strategy not only achieved maximal antioxidant effects but also promoted energy metabolism restoration in the liver (Figure 11D) [110].

#### 5.2.2 Liver Fibrosis

Liver fibrosis is the result of chronic inflammation and the accumulation of damaged cells induced by viral infections, metabolic disorders, and autoimmune diseases. Liver fibrosis is characterized by fibrous scar tissue mainly composed of extracellular matrix (ECM) proteins, primarily collagen types I and III [111]. Unfortunately, clinical drug interventions have achieved only limited therapeutic efficacy. A consensus has emerged that liver fibrosis is reversible in both clinical patients and experimental animal models, owing to effective treatment of underlying chronic liver diseases such as hepatitis B virus (HBV) or hepatitis C virus (HCV) infection and alcohol abuse [112]. The key molecular mechanisms leading to liver fibrosis are the activation of HSCs. More than 90% of collagen‐producing cells are HSCs and activated portal fibroblasts [113]. Therefore, desirable therapeutic effects can be achieved by nanozymes through maintaining HSCs in a deactivated state.

**FIGURE 12:**
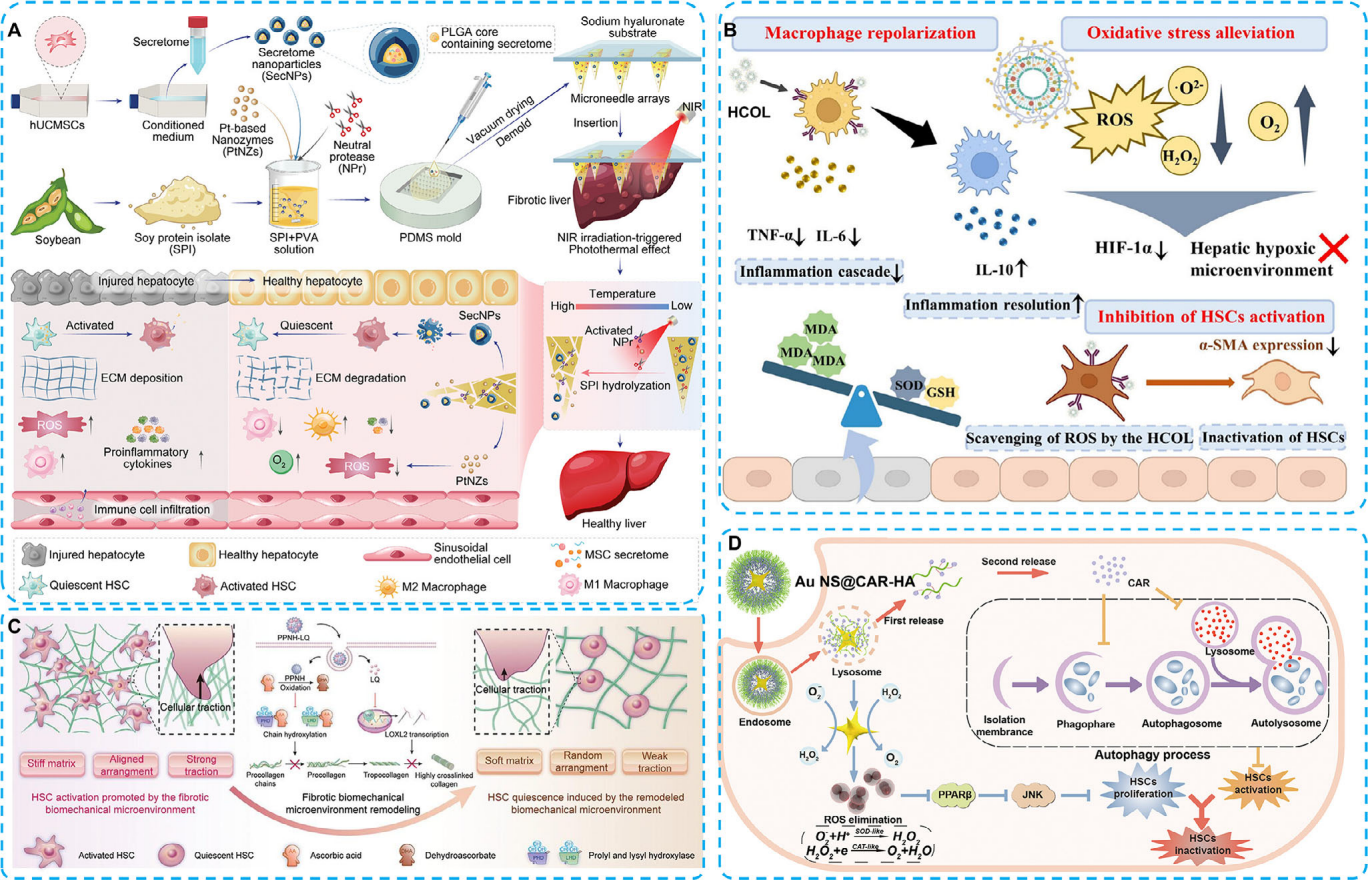
(A) Schematic illustration of the fabrication and implantation of NIR‐responsive MN arrays containing hUCMSC‐derived secretome‐encapsulated core–shell nanoparticles and versatile nanozymes to alleviate liver fibrosis. Reproduced with permission [119]. Copyright 2024 The Author(s). Advanced Science is published by Wiley‐VCH GmbH. (B) Illustrative diagram depicting the preparation of HCOL and its operational mechanism in the treatment of liver fibrosis. Reproduced with permission [123]. Copyright 2024 American Chemical Society. (C) Illustration of PPNH‐LQs mediated treatment for hepatic fibrosis by remodeling the fibrotic biomechanical microenvironment. Reproduced with permission [127]. Copyright 2024 Wiley‐VCH GmbH. (D) Mechanism of Au NS@CAR‐HA treating hepatic fibrosis by removing ROS and inhibiting autophagy. Reproduced with permission [130]. Copyright 2021 Elsevier B.V.

Platinum‐based nanozymes integrated with carbon nanodots (CNDs) (Pt@CNDs) have been demonstrated to exhibit excellent SOD‐ and CAT‐like catalytic properties in regulating ROS and reducing inflammation in CCl_4_‐induced liver injury [114]. Stem cell therapy has garnered interest in tissue repair and organ regeneration [115]. To avoid potential immunological rejection, stem cell secretome‐based cell‐free therapy has shown promising therapeutic effects [116]. The inescapable challenge is the identification of an ideal delivery vector for optimal drug accumulation at injury sites. The microneedle (MN) array is a burgeoning drug delivery platform due to the fact that it is painless, efficient, biocompatible, and biodegradable [117, 118]. Herrin, Xu, and co‐workers designed an innovative smart MN array for the delivery of multiple therapeutic agents in liver fibrosis treatment. The MNs were prepared using a mixture of soy protein isolate (SPI) and polyvinyl alcohol (PVA), with a heat‐induced structure unfolding method. This process encapsulated stem cell secretome nanoparticles (SecNPs), Pt‐based nanozymes (PtNZs), and neutral protease (NPr). After in situ transplantation, the MNs were exposed to near‐infrared (NIR) irradiation, which reached the temperature required for NPr activation. The NPr further catalyzed the degradation of MNs for the controlled release of PtNZ and SecNPs to the fibrotic liver. The elimination of ROS and the degradation of the ECM ultimately maintained HSCs in a quiescent state and reduced inflammation (Figure 12A) [119].

The multi‐metallic catalyst systems reveal better catalytic activity and catalyst durability than single pure metals, including Ag and Pt [120]. Jing et al. developed another Pt‐based nanozyme formulated with Ag. The Ag@Pt nanozymes were modified with hyaluronic acid (HA) and loaded with nilotinib (NIL) to relieve liver fibrosis. The HA modification on Ag@Pt‐NIL/HA nanotriangles (APNH NTs) provided the targeting strategy for HSCs via CD44 expression on the surface. The Ag@Pt delivery platform possesses excellent SOD and CAT‐like activities in ROS elimination. In addition, NIL released from the APNH NTs induced collagen degradation by downregulating TIMP‐1. This enlightening strategy provides a promising approach for the treatment of liver fibrosis [121].

Cerium oxide nanoparticles (CeO_2_ NPs) are potential nanozymes possessing SOD‐like and CAT‐like catalytic activities, which are expected to eliminate the ROS produced in the liver [122]. The cycling between Ce^3+^ and Ce^4+^, through the binding oxygen atoms, gives CeO_2_ NPs the ability to scavenge ROS. Yang et al. designed another HA‐targeting liposome delivery system (HCOL), which loaded oleanolic acid (OA) and was surface‐modified with HA/CeNPs. Efficient accumulation can be achieved in both KCs and HSCs depending via CD44 receptor‐mediated phagocytosis. The release of OA in macrophages promotes macrophage polarization from M1 to an M2 phenotype. This ingenious nanoplatform combines multiple therapeutic effects, including ROS elimination, M2 phenotype polarization, and HSC deactivation in liver fibrosis treatment (Figure 12B) [123].

The physiological activities of HSCs and the stable mechanical characteristics of the ECM maintain the balance of mechanical homeostasis and support the normal functions of liver resident cells [124, 125, 126]. Apart from scavenging ROS, nanozymes also have the potential to inhibit collagen production. Wang and co‐workers utilized the ascorbic acid‐oxidase (AAO) catalytic activity of Pt@Pd‐based hedgehogs (PPNHs) to consume intracellular ascorbic acid, which was indispensable for collagen synthesis. Liquiritigenin (LQ), an inhibitor of YAP‐mediated transcription of lysyl oxidase‐like 2 (LOXL2), was encapsulated in the PPNHs (PPNHs‐LQs). In vivo results revealed the elimination of overproduced collagen and further regulation of the biomechanical microenvironment. PPNHs‐LQs therapy remodeled mechanical homeostasis, inhibited activation of HSCs, and regressed liver fibrosis (Figure 12C) [127]. Research showed that the inhibition of autophagy exhibited a promising therapeutic effect on the suppression of HSCs [128, 129]. Li's group applied an autophagy‐inhibiting therapy to regulate the activation of HSCs. They utilized Au nanozymes (star‐like gold) to control ROS production, depending on their SOD‐ and CAT‐like catalytic activities. Carvedilol (CAR), an autophagy inhibitor regulating lysosomal pH, and HA were coated on the surface of Au nanozymes (Au NS@CAR‐HA). With this strategy, the activation and proliferation of HSCs were effectively limited, and the progression of liver fibrosis was prevented (Figure 12D) [130]. Taken together, these approaches demonstrate that nanozyme‐based therapies can target multiple pathogenic mechanisms in liver fibrosis, including ROS scavenging, collagen suppression, and autophagy regulation, thereby providing a comprehensive and synergistic therapeutic framework.

#### 5.2.3 Hepatic Ischemia‐Reperfusion Injury

HIRI is a common and inevitable result of hepatic trauma, liver resection, or liver transplantation. Severe HIRI accounts for approximately 10% of early graft failures and increases the risk of postoperative complications, thereby compromising long‐term graft survival [131, 132, 133]. HIRI comprises two stages: the ischemia stage and the reperfusion stage. During the liver surgical procedures, ischemia, or insufficient oxygen supply, represents a critical stage [134]. However, a large amount of oxygen is essential for basic metabolic processes and biochemical reactions. A reduced blood supply can therefore readily induce liver injury [135]. The reperfusion stage begins with the restoration of blood flow, triggering the complement cascade and causing secondary damage [136, 137]. Numerous risk factors are involved in the pathogenesis of HIRI, including the release of pro‐inflammatory cytokines, metabolic and oxidative stress, and the activation of KCs [138]. The occurrence of ROS creation and oxidative stress occurring in KCs, LSECs, and hepatocytes is a primary mechanism underlying HIRI [139]. Since excessive ROS production and subsequent oxidative stress promote the development of HIRI, antioxidant therapy is considered a promising strategy to attenuate it during liver transplantation and related surgical procedures. [140].

**FIGURE 13:**
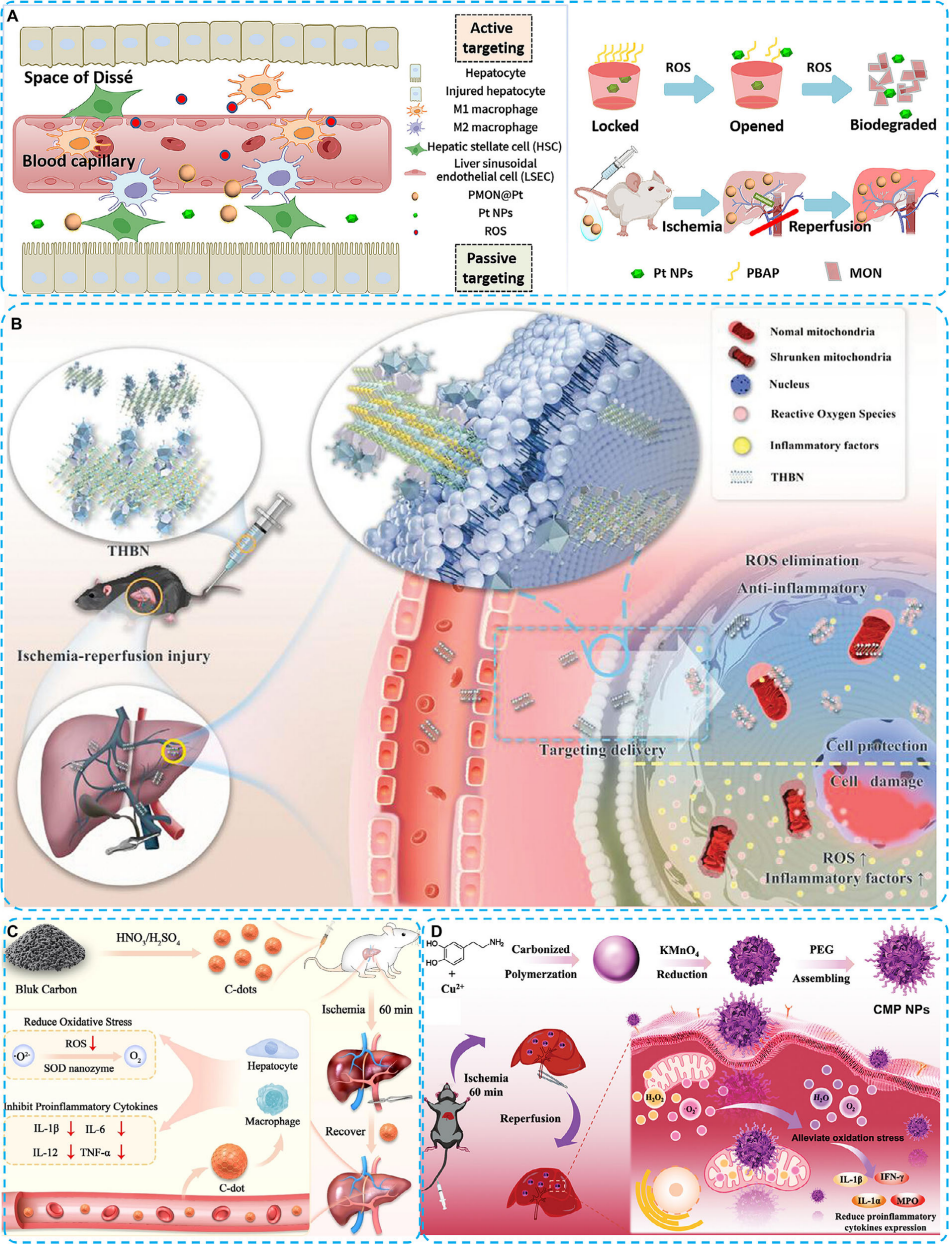
(A) Schematic illustration of the protective effects of PMON@Pt on HIRI therapy. Reproduced with permission [144]. Copyright 2025 Elsevier B.V. (B) Schematic illustration of robust ROS scavenging ability of THBN that endows it with excellent ability to prevent HIRI. Reproduced with permission [146]. Copyright 2025 Wiley‐VCH GmbH. (C) Illustration depicting the production of C‐dots nanozymes and their therapeutic application in HIRI. Reproduced with permission [152]. Copyright the Author(s) 2023, Journal of Nanobiotechnology published by BMC, Springer Nature. (D) Schematic diagram of hepatic IRI treatment with CMP NPs. Reproduced with permission [153]. Copyright 2024 Wiley‐VCH GmbH.

Platinum nanoparticles (Pt NPs) are universally acknowledged as outstanding nanozymes, mimicking SOD‐, CAT‐, and POD‐like catalytic activities [141, 142, 143]. Pt NPs exhibit superior chemical stability and catalytic efficiency compared with other exogenous antioxidants. However, challenges remain in Pt NP‐based therapy, including potential cytotoxicity and unexpected accumulation in organs. To address these issues, Zhou et al. developed a novel liver‐targeted drug delivery system based on mesoporous organosilica nanoparticles (MONs) to minimize the adverse effects of Pt nanozymes (MON@Pt). Phenylboronic acid pinacol ester (PBAP) was employed as a surface functional group, enabling sensitive responsiveness to H_2_O_2_ (PMON@Pt). Under oxidative stress during HIRI, the cleavage of tetrasulfide bonds and PBAP accelerated the degradation of PMON@Pt, thereby releasing the loaded Pt NPs. Moreover, in a rat HIRI model, PMON@Pt exhibited remarkable accumulation in hepatocytes and KCs, effectively scavenging ROS and reducing inflammatory cytokine levels. This work provided an ideal nanozyme delivery platform, featuring efficient accumulation and favorable therapeutic effects in the prevention and treatment of HIRI (Figure 13A) [144].

As 2D nanomaterials, Ti_3_C_2_ nanosheets (MXene) also exhibit superior CAT‐like catalytic activity, broadly eliminating ROS generated during the HIRI process. Zheng and co‐workers synthesized Ti_3_C_2_ nanosheets decorated with adeno‐associated virus serotype 8 (AAV8) via tannic acid (TA) linkers. They regarded this universal strategy as a Trojan horse‐like biohybrid nanozyme (THBN) that specifically targets the liver. AAV8, a well‐established gene therapy vector, has been utilized in patients with severe hemophilia B (NCT00979238), achieving sustained clinical benefit [145]. With such modification, the 2D Ti_3_C_2_ nanosheets demonstrated high accumulation in the liver and provided effective therapeutic benefits against liver I/R injury. ROS elimination and the anti‐inflammatory effects induced by Ti_3_C_2_ nanozymes prevented disease progression (Figure 13B) [146]. AAV8 is considered a highly efficient delivery vector for liver gene therapy. However, concerns remain regarding its immunogenicity, batch‐to‐batch quality control, and the cost of large‐scale production. We believe that the development of AAV or other nanoparticles will ultimately lead to an ideal drug delivery system featuring biosafety, high efficiency, and low cost for the treatment of liver diseases.

Carbon‐based materials include carbon nanotubes, carbon nitride, carbon dots (C‐dots), and fullerenes [147, 148, 149]. Among them, C‐dots nanozymes exhibit higher SOD‐like activity than Cu nanozymes in scavenging free radicals [150, 151]. Geng et al. employed the SOD‐like activity of C‐dots to explore their therapeutic effects against HIRI. First, they demonstrated that C‐dots could prevent apoptosis in L‐02 cells under oxidative stress and inflammatory challenge. In a mouse model of HIRI, C‐dots nanozymes successfully eliminated ROS and attenuated inflammation, resulting in significant therapeutic effects against liver I/R injury. Furthermore, transcriptome sequencing results indicated that C‐dots regulated GSH metabolism and the TGF‐beta signaling pathway, thereby enhancing therapeutic effectiveness (Figure 13C) [152].

Another potential substitute for natural enzymes in antioxidant therapy is the ultrasmall copper nanozymes (Cu_us_) [51]. This single‐atom Cu_us_ catalyst possesses CAT‐like and SOD‐like catalytic activities, with high stability and low cost. Lin and co‐workers designed a novel nanozyme system, in which Cu_us_‐pC was carbonized within MnO_2_ (Cu_us_‐pC@MnO_2_) and further modified with PEG, yielding Cu_us_‐pC@MnO_2_@PEG nanoparticles (CMP NPs). In both in vivo and in vitro studies, the CMP NPs exhibited multienzyme‐like activities, effectively removing endogenous ROS and alleviating mitochondrial oxidative stress. Compared with the clinical antioxidant drug NAC, CMP NPs achieved similar hepatoprotective effects at a lower dosage. This work demonstrated a promising nanozyme‐based therapy with potential for the clinical treatment of HIRI (Figure 13D) [153].

Stem cell therapy is considered an alternative approach for repairing injured hepatocytes during HIRI. Mesenchymal stem cells (MSCs) exhibit the potential to differentiate into specific cell types and mediate pleiotropic signaling [154]. In a HIRI rat model, bone marrow‐derived MSCs successfully inhibited apoptosis and promoted the regeneration of hepatocytes [155]. As mentioned above, a major challenge of stem cell therapy lies in the excessive production of endogenous ROS [103]. PB nanoparticles are predominantly applied in magnetic resonance imaging (MRI) because of their biocompatibility [156, 157]. PB nanoparticles also possess remarkable POD‐, CAT‐, and SOD‐like multienzyme activities, which can effectively scavenge ROS [158]. Therefore, Sahu et al. utilized PB nanoparticles, an ideal nanomaterial, to preserve the functionality of MSCs. In the PB combined with MSCs (PB‐MSC) therapy, HIRI‐induced ROS and oxidative stress were significantly reduced. Furthermore, PB nanozymes loaded on MSCs enhanced the secretion of anti‐inflammatory cytokines, thereby remodeling the liver microenvironment during I/R injury [159].

In summary, nanozymes show strong potential for liver tissue repair by scavenging ROS, reducing inflammation, and regulating fibrotic activation. When combined with advanced delivery systems or stem cell therapies, they further enhance hepatocyte regeneration and microenvironment remodeling. These strategies highlight nanozymes as versatile candidates for managing ALF/ALI, fibrosis, and HIRI, paving the way for clinical translation.

### 5.3 Hepatocellular Carcinoma

Liver disease causes about 2 million deaths annually [82]. The majority of these deaths are primarily attributable to the progression of HCC. Liver cancer is the fourth leading cause of malignant tumor‐related death, with a 5‐year survival of 18% [160, 161]. The incidence of liver cancer is projected to exceed 1 million cases by 2025, with HCC representing the predominant subtype and accounting for approximately 90% of cases [162]. Currently, about half of HCC patients receive first‐line treatment with sorafenib or lenvatinib. Immune‐checkpoint inhibitors (ICIs) have ushered in a new era of cancer immunotherapy. Pembrolizumab and nivolumab, two ICIs targeting programmed cell death‐1 (PD‐1), have been approved by the FDA for the treatment of advanced HCC in patients who previously failed on sorafenib therapy [163, 164]. Furthermore, nivolumab combined with ipilimumab (a CTLA‐4 monoclonal antibody) and the combination of atezolizumab (anti‐PD‐L1) with bevacizumab (anti‐VEGF) have also been approved as second‐line and first‐line treatments, respectively, for advanced HCC [165, 166]. However, despite these advances, only 20–30% of HCC patients respond to immunotherapy [167]. These clinical therapies focus their effects at the cell level (reducing tumor cells or activating T cells). In contrast, nanozymes therapeutic applications provide additional potential for alleviating the pH levels and hypoxia within the TME. Therefore, the role of the liver TME in HCC tumorigenesis should be carefully considered when developing new therapeutic strategies.

ROS play diverse roles as secondary messengers in a manner defined by their variable concentrations, distributions, and durations [168]. One significant characteristic of cancer cells is their increased ROS levels. Excessive ROS initiates tumorigenesis and promotes tumor progression [169]. When ROS accumulation exceeds a critical threshold, their pro‐carcinogenic roles in promoting proliferation and invasion switch to antitumor effects. This shift is primarily mediated through regulated cell death (RCD), including apoptosis, necroptosis, and ferroptosis [168]. Therefore, to precisely modulate ROS levels, nanozymes should suppress pro‐carcinogenic effects while maximizing antitumor effects by generating excessive ROS in tumor cells and minimizing off‐target effects in healthy tissues. Elevated ROS levels lead to DNA damage, resulting in genetic mutations that may trigger cancer. Conversely, sustained ROS production can also induce antitumor responses by promoting apoptosis, necroptosis, or ferroptosis in cancer cells. Nanozymes hold great promise for catalyzing ROS generation, depleting endogenous antioxidants, and enhancing antitumor responses. Moreover, to overcome the tumor‐tolerant microenvironment, nanozymes can enhance the efficacy of combination therapies with radiotherapy, chemotherapy, photodynamic therapy, and immunotherapy.

**FIGURE 14:**
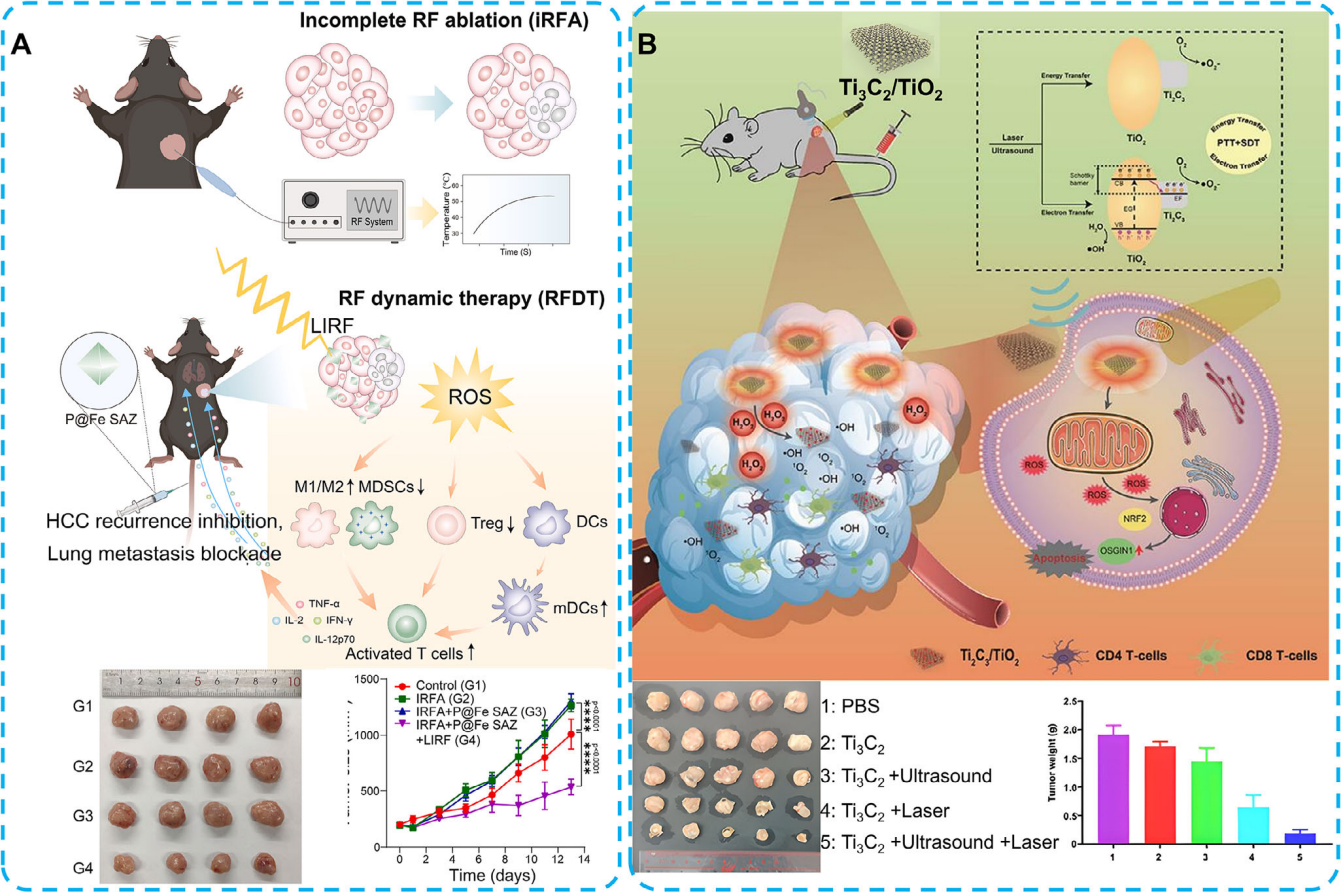
(A) Schematic illustration of iRFA establishment and action principles and targets of low‐temperature RFDT to mitigate iRFA‐aggravated immunosuppression against tumor growth and lung metastasis. Reproduced with permission [173]. Copyright 2025 Elsevier Ltd. (B) Schematic diagram of in situ grown nanocrystal TiO_2_ on 2D Ti_3_C_2_ nanosheets enabling photo‐sonodynamic activity and enhanced tumor ablation via immunotherapy. Reproduced with permission [180]. Copyright 2024 The Author(s). Dove Medical Press Ltd.

For elderly or debilitated patients, radiofrequency ablation (RFA) is often regarded as a primary therapeutic option due to its minimally invasive nature [170]. However, incomplete radiofrequency ablation (iRFA) is often unavoidable. Research has indicated that iRFA‐induced inflammation and immune dysregulation may promote tumor progression [171, 172]. Herein, Fang and co‐workers developed an iron (Fe)‐based single‐atom nanozyme with PEG modification (P@Fe SAZ). This approach achieved radiofrequency dynamic therapy (RFDT) under low‐intensity radiofrequency (LIRF) irradiation for the first time. The low‐temperature (<42°C) RFDT under LIRF irradiation (2W‐15s) successfully produced ROS, driving the polarization of tumor‐associated macrophages (TAMs) from the immunosuppressive M2 phenotype to the antitumor M1 phenotype. The iRFA‐induced immunosuppression was reversed, accompanied by the secretion of anti‐tumor cytokines, including interleukin‐2 (IL‐2), interleukin‐12p70 (IL‐12p70), TNF‐α, and interferon‐γ (IFN‐γ). In this approach, ROS produced by P@Fe SAZ remodeled the immunosuppressive TME, providing an innovative therapeutic strategy for HCC (Figure 14A) [173].

Photothermal therapy (PTT) exhibited effective therapeutic effects against malignant cancers [174, 175]. However, its clinical application is limited by poor tumor tissue penetration and the risk of systemic phototoxicity [176, 177]. Therefore, the exploration of novel nanomaterials or therapeutic approaches continues. Sonodynamic therapy (SDT) has emerged as a novel noninvasive therapeutic modality with high tissue‐penetrating capacity. In this approach, sonosensitizers can be activated by ultrasound to generate ROS, thereby inducing apoptosis in cancer cells [178]. Yu, Huang, Nong, and colleagues developed Ti_3_C_2_ nanozymes, a 2D MXene material, which possess outstanding photothermal properties and strong absorption in the NIR‐I and NIR‐II regions [179]. Furthermore, the titanium atoms in Ti_3_C_2_ can serve as nucleation sites to generate TiO_2_ during oxidation reactions, ultimately forming a Ti_3_C_2_/TiO_2_ heterostructure. Therefore, Ti_3_C_2_ functions as a multifunctional nanoplatform for both PTT and SDT in HCC treatment. In mouse models, the POD‐like activity of Ti_3_C_2_ enhanced ROS generation within the TME. Moreover, the construction of the Ti_3_C_2_/TiO_2_ heterostructure provided an excellent opportunity for combined sonodynamic and photothermal therapies, effectively reversing the TME from an immunologically “cold” to a “hot” state (Figure 14B) [180].

The five‐year recurrence rate of HCC patients after surgery is approximately 70% [162]. Precise prognostic diagnostic methods are still urgently needed for clinical practice. Yan's group previously developed the first ferritin‐based nanozyme with outstanding POD‐like activity. Human heavy‐chain ferritin and Fe_3_O_4_ NPs were biomimetically synthesized into a magnetoferritin (M‐HFn) nanozyme, which enabled targeted delivery to transferrin receptor 1 (TfR1)‐positive tumor tissues [181]. However, the POD‐like activity of Fe_3_O_4_ is inferior to that of natural HRP [3]. To overcome this limitation, their team subsequently developed an improved strategy for constructing ferritin‐based nanozymes. Co_3_O_4_ NPs were demonstrated to exhibit higher POD‐like activity than Fe_3_O_4_ NPs [182, 183]. Moreover, the SP94 peptide was reported as a specific targeting ligand for HCC cells [184], and was widely utilized for HCC diagnosis and therapy. Therefore, they designed a novel HCC‐targeted HccFn (Co_3_O_4_) nanozyme, which was able to distinguish HCC tissues in 424 clinical specimens with a sensitivity of 63.5% and a specificity of 79.1%. This ferritin‐based Co_3_O_4_ nanozyme provided a promising approach for the prognostic diagnosis of HCC [185].

HCC remains a major global health burden with high recurrence rates and limited therapeutic responses. Nanozyme‐based strategies show great potential in both diagnosis and therapy by modulating ROS, reshaping the TME, and enabling targeted treatment, thereby offering promising avenues for improving patient outcomes.

### 5.4 Other Liver Disease

#### 5.4.1 Hepatitis C Virus

HCV infection remains a major global public health burden. Over 15 million people were diagnosed with HCV worldwide between 2015 and 2019 [186]. Currently the clinical anti‐chronic HCV therapies are shifting from interferon‐α combined with ribavirin to direct acting antivirals (DAAs). The sustained virological response of DAAs reaches 90% in HCV patients. The appearance of oral DAAs has greatly improved the therapeutic efficacy in HCV treatments. However, most of DAAs fail in patients with decompensated liver cirrhosis, due to the impaired metabolic function of hepatocytes.

**FIGURE 15:**
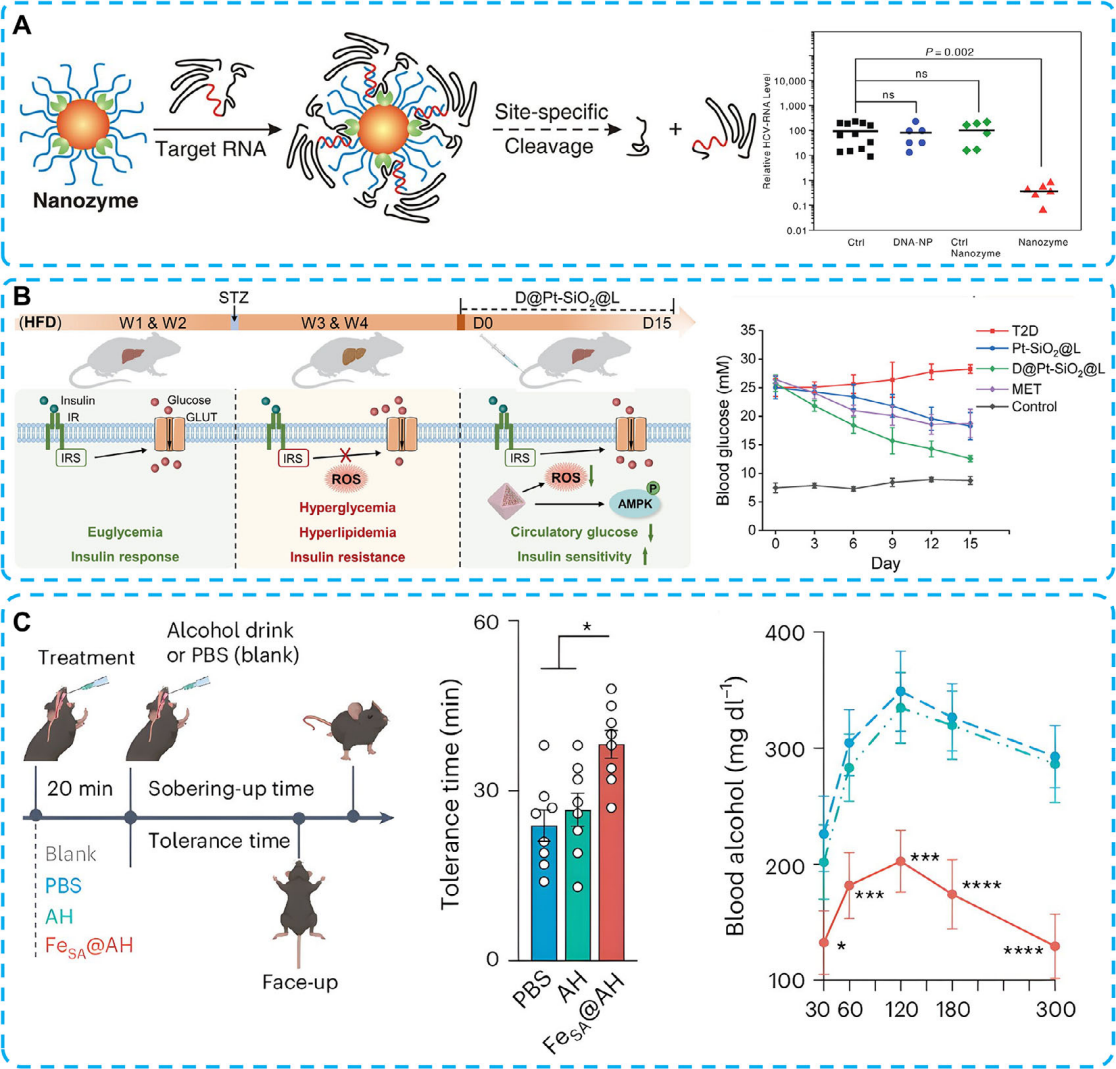
(A) Schematic representation describing the design and the anti‐HCV function of Au‐based nanozyme. Reproduced with permission [189]. Copyright 2012 National Academy of Sciences. (B) Biodegradable platinum‐silica nanoshells carrying an uncoupler targeting the liver to reverse insulin resistance in type 2 diabetes. Reproduced with permission [194]. Copyright 2023 American Chemical Society. (C) Schematic of the treatment of acute alcohol intoxication model with FeSA@FibBLG nanozymes. Reproduced with permission [199]. Copyright 2024 Springer Nature.

HCV is a positive‐sense RNA virus comprising six major genotypes and numerous subtypes [187]. Wang et al. utilized Au‐based nanozymes to mimic RNA‐cleavage activity, thereby enabling RNA interference (RNAi) pathways [188]. The endoribonuclease and DNA oligonucleotides complementary to the HCV RNA sequence (nucleotides 322–339) were conjugated to the Au‐based nanozymes. In an HCV mouse model, the anti‐HCV nanozymes cleaved the HCV RNA segment in a sequence‐specific manner. With this strategy, the adverse effects of Au‐based RNAi therapy are greatly avoided, reducing the unnecessary burdens in healthy tissues. These findings demonstrated the potential of nanozymes as an antiviral therapy against HCV and other viral hepatitis infections (Figure 15A) [189].

#### 5.4.2 Type 2 Diabetes

Diabetes has become a major global public health concern, with over 537 million people diagnosed worldwide [190]. Type 2 diabetes (T2D) accounts for over 90% of all diabetes cases and is primarily caused by insulin resistance [191]. The primary risk factors of T2D include excessive intake of a high‐fat diet, resulting in inflammation and oxidative stress‐induced insulin resistance [192, 193]. Therefore, scavenging ROS in the liver and restoring hepatic insulin sensitivity have become an ideal strategy for the treatment of T2D.

Zhang and co‐workers utilized a hollow SiO_2_ core and a phospholipid bilayer to encapsulate Pt nanomaterials and the mitochondrial uncoupler DNPME (D@Pt‐SiO_2_@L). This surface modification improved the liver‐targeted delivery efficiency of Pt nanozymes and DNPME. The ultrasmall Pt NPs were released into hepatocytes, where they eliminated ROS, while DNPME inhibited ROS production in the mitochondrial electron transport chain. Meanwhile, DNPME also activated the AMPK signaling pathway to reverse insulin resistance, thereby restoring glucose metabolism in the liver (Figure 15B) [194].

In another therapeutic strategy for T2D, Li's group designed AuCePt porous hollow cascade nanozymes (AuCePt PHNs) as a delivery system. Lactobionic acid (LA) modification was employed to target hepatocytes through ASGPR‐mediated internalization. Disulfiram (DSF), an inhibitor of nuclear factor‐κB (NFκB), was loaded into the NPs (AuCePt PHNs‐LA@DSF) for metabolic regulation in hepatocytes. Their results showed that AuCePt PHNs scavenged ROS and inhibited oxidative stress through SOD‐ and CAT‐like catalytic activities. The nanozyme system successfully reversed insulin resistance by activating the IRS‐1/AKT pathway and inhibiting the FOXO‐1/PEPCK pathway in hepatocytes. In general, AuCePt PHNs‐LA@DSF demonstrated potential efficacy in T2D treatment by controlling blood glucose levels, improving insulin sensitivity, and reducing lipid deposition [195].

#### 5.4.3 Acute Alcohol‐Induced Liver Injury

Alcohol abuse has become a major global health risk. In fact, harmful alcohol consumption has caused approximately 3 million deaths and 132.6 million disability‐adjusted life years [196]. Current therapies rely solely on endogenous enzymes, providing relief from nausea and headaches but failing to address fatigue and chronic alcoholism. Nanocomplexes have been proven to enhance the natural enzymes HRP and GOx, thereby preventing alcohol intoxication and accelerating alcohol metabolism [197, 198]. However, commercially available enzymes face a major limitation: acetaldehyde accumulation, a more hazardous intermediate resulting from insufficient catalytic activity. Therefore, nanozymes with high catalytic efficacy, low cost, and outstanding physicochemical stability have attracted considerable scientific interest in therapeutic applications.

Mezzenga et al. designed a single‐site iron‐anchored β‐lactoglobulin (BLG) amyloid fibril (Fe_SA_@FibBLG) nanozyme system, which exhibited excellent catalytic activity. This biomimetic nanozyme alleviated alcohol‐induced damage when administered orally. FeSA@FibBLG mimicked the coordination structure of HRP and achieved remarkable POD‐like activity by catalyzing alcohol oxidation. In vivo experiments showed that the gelatinous nanozyme could withstand the digestive process and reduce blood alcohol levels in alcohol‐exposed mice. Moreover, its high POD‐like catalytic efficacy prevented the accumulation of acetaldehyde. This work offers promising insight into alcohol detoxification, with potential benefits for liver protection (Figure 15C) [199].

Most inorganic materials exhibit poor biodegradability, resulting in prolonged retention in the liver. Metal or metal oxide nanozymes should be designed to possess high long‐term stability. This is crucial, as ionic forms of certain metals can induce hepatic cytotoxicity. In chronic liver disease therapy, the duration of catalytic activity influences the required dosage, thereby impacting safety. Excessive ROS scavenging disturbs the normal functions of organs. Thus, the ideal design of future intelligent nanozymes for chronic liver diseases should include a controllable switch to regulate catalytic duration. Moreover, they should have distinctive catalytic performance within the different stages of liver disease. It is not necessary to maintain the catalytic activity of the nanozymes throughout the entire course of the disease.

In summary, nanozymes demonstrate versatile applications in managing various liver‐related diseases beyond MAFLD, liver tissue repair, and HCC. They provide innovative strategies for antiviral therapy against HCV, improve insulin sensitivity and glucose metabolism in T2D, and facilitate alcohol detoxification with hepatoprotective effects (Table 2). These studies highlight the broad therapeutic potential, especially regarding the metabolic regulatory roles of nanozymes in liver disease treatment. In spite of dramatic improvements in enhancing the therapeutic efficacy, nanozymes still face concerns regarding the long‐term biocompatibility of nanomaterials for further clinical use.

**TABLE 2:** Applications of Nanozymes in Liver Disease Therapy.

Liver diseases	Nanozymes	Catalytic activity	Targeting cell	Therapeutic mechanism	Reference
MAFLD	Ta_4_C_3_	SOD‐like	Hepatocyte and macrophage	Scavenging ROS; suppressing TNF‐α, IL‐6, and IL‐1β	[95]
	nCeO_2_	SOD‐ and CAT‐like	Hepatocyte	Promote IL‐4, IL‐10, and TGF‐β	[96]
	H‐CeO_2_	SOD‐ and CAT‐like	Hepatocyte	Scavenging ROS	[97]
ALF/ALI	Au NCs	SOD‐ and CAT‐like	Hepatocyte and macrophage	Cell proliferation, ROS elimination, and anti‐inflammation	[104]
	SAuPTB	SOD‐ and CAT‐like	Hepatocyte	Hepatocytes protection and inflammation regulation	[108]
	TBP@CeO_2_	SOD‐ and CAT‐like	Hepatocyte	•OH elimination and restore mitochondrial function	[110]
Liver fibrosis	Pt@CNDs	SOD‐ and CAT‐like	HSC	Reducing mitochondria ROS	[114]
	SecNPs and PtNZs	SOD‐like	HSC and macrophage	HSCs are quiescent, and M2‐macrophages polarization	[119]
	Ag@Pt‐ NIL/HA	SOD‐ and CAT‐like	HSC	Inhibition of HSCs activation and remodeling of the liver fibrosis microenvironment	[121]
	HA/CeNPs	SOD‐ and CAT‐like	HSC and macrophage	Macrophage repolarization, oxidative stress alleviation, and inhibition of HSCs activation	[123]
	Au NS@ CAR‐HA	SOD‐ and CAT‐like	HSC	Regulating the proliferation and activation of HSCs	[130]
HIRI	PMON@Pt	SOD‐ and CAT‐like	KC and hepatocyte	Suppression of inflammatory cascades; liver protection.	[144]
	THBN (Ti_3_C_2_)	CAT‐like	Hepatocyte	Mitigating inflammation and oxidative stress, and inhibiting hepatocellular apoptosis	[146]
	C‐dots	SOD‐like	Macrophage and hepatocyte	Reduce oxidative stress and inhibit proinflammatory cytokines	[152]
	Cu_us_‐pC @MnO_2_@PEG	SOD‐ and CAT‐like	Hepatocyte	Alleviate oxidation stress and reduce proinflammatory cytokine expression	[153]
	PB‐MSC	SOD‐like	KC and hepatocyte	Oxidative stress management, efficient release of cytokines/growth factors, and immunomodulatory functions	[159]
HCC	P@Fe SAZ	POD‐like	HCC cells	ROS remodels the iRFA‐potentiated immunosuppressive microenvironment	[200]
	Ti_3_C_2_/TiO_2_	POD‐like	HCC cells	ROS regulates apoptosis of HCC and enhances immune infiltration of cytotoxic T cells	[180]
	HccFn (Co_3_O_4_)	POD‐like	HCC cells	Ferritin‐based detection of HCC samples	[185]
HCV	Au	Nuclease‐like	Hepatocyte	Cleave the HCV RNA segment in a sequence‐specific manner	[189]
T2D	D@Pt‐SiO_2_@L	SOD‐ and CAT‐like	Hepatocyte	Reversing hepatic insulin resistance through long‐term ROS scavenging	[194]
	AuCePt PHNs	SOD‐ and CAT‐like	Hepatocyte	Scavenging ROS; anti‐oxidant stress	[195]
Acute alcohol‐induced liver injury	Fe_SA_@ FibBLG	POD‐like	Hepatocyte	Selectively catalyze alcohol oxidation into acetic acid	[199]

MAFLD: metabolic dysfunction‐associated fatty liver disease; ALF/ALI: acute liver failure/acute liver injury; HIRI: hepatic ischemia‐reperfusion injury; T2D: type 2 diabetes; HCC: hepatocellular carcinoma; HCV: hepatitis C virus; SOD: superoxide dismutase; CAT: catalase; POD: peroxidase; ROS: reactive oxygen species; KC: Kupffer cell; HSC: hepatic stellate cell.

## 6 Artificial Intelligence (AI) Drives the Revolution in Nanozymes

AI is an emerging field of computer science that develops intelligent systems endowed with the capabilities of perception, pattern analysis, and problem‐solving. Machine learning is a specific application of AI, using mathematical models by analyzing massive datasets to predict potential high‐activity nanozymes [201]. This approach is particularly suitable for designing nanozymes from a wide range of nanomaterials and combinations, and for optimizing their catalytic efficiency and selectivity [202]. Beyond predictive modeling, AI facilitates the analysis of intricate catalytic reaction pathways, offering insights into substrate recognition mechanisms and catalytic efficiency. The integration of AI with multiscale simulations and experimental optimization has also accelerated research workflows, significantly shortening development timelines. Furthermore, AI‐driven intelligent synthesis systems dynamically design experimental routes, enhancing both the success rate and efficiency of nanozyme synthesis while providing adaptability for complex environmental applications [203].

**FIGURE 16:**
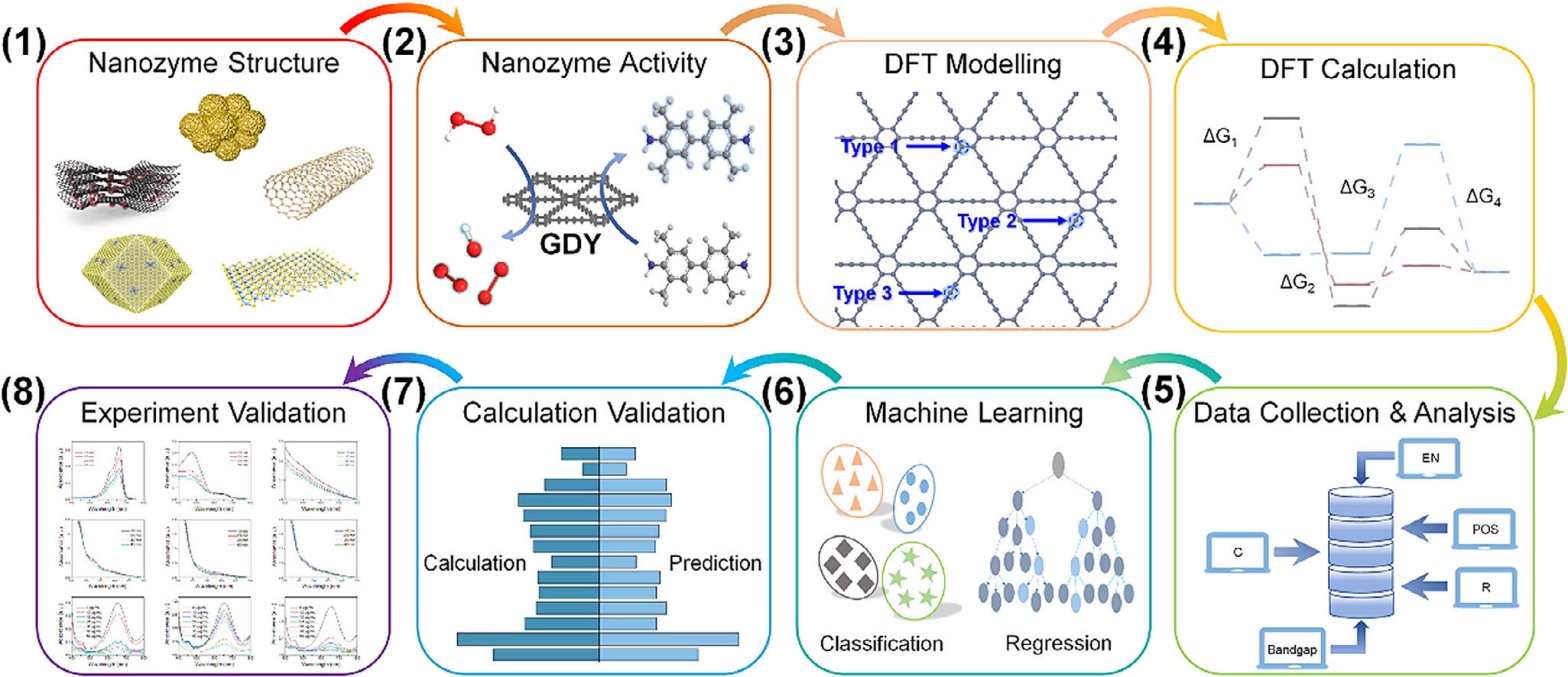
Schematic illustration of the workflow for the machine learning‐assisted GDY‐based nanozyme discovery. GDY: graphdiyne DFT: density functional theory. Reproduced with permission [206]. Copyright 2022, American Chemical Society.

To develop novel machine learning‐designed nanozymes with high catalytic performance, the initial step is to construct a multi‐source database. This database should include standardized and specific information on nanozymes, such as size, morphology, surface modification, composition, and crystal structure. These key features constitute a standardized database for machine learning, enabling the prediction of nanozymes with high catalytic performance. For example, material information on nanozymes collected from 4159 papers was utilized to construct such a database [203]. To ensure data quality, the raw data must undergo strict standardization, including eliminating outliers caused by experimental errors, normalizing the catalytic reaction rate at different temperatures, electrolyte concentrations, and pH values across different studies, and constructing a standard database for nanozymes [204]. Subsequently, regression models were built to establish a relationship between the database and nanozyme catalytic activity, leading to accurate predictions of catalytic performance. Moreover, the integration of quantum mechanics/molecular mechanics methods with machine learning algorithms has enabled the identification of optimal designs for multienzyme‐like nanozymes. Ultimately, Zhu and Sui et al. created and verified CuMnCo_7_O_12_, a highly active multienzyme‐like nanozyme with enhanced POD‐, OXD‐, CAT‐, and sustained SOD‐like catalytic activities [205]. To investigate the optimal POD‐like catalytic ability of nonmetal atom‐doped graphdiyne (GDY), Zhu and Sui's group applied the extreme gradient boosting (XGB) algorithm to mine the relationship between model parameters and the maximum energy barrier (R^2^ > 78%) or the maximum energy‐consuming step (accuracy > 65%) using data from all nonmetal‐atom‐doped GDYs. Moreover, they confirmed that three predicted products exhibited the expected catalytic properties. This study indicated that machine learning can be effectively utilized to guide the design of nanozymes through density functional theory‐based computational screening (Figure 16) [206]. Thus, machine learning provides powerful computational approaches to address challenges posed by the vast material space and complex reaction mechanisms in nanozyme design.

Despite these achievements, traditional experimental approaches remain limited by long timelines, high costs, and inefficient trial‐and‐error processes, hindering the tailored design of nanozymes for complex applications. Recent advances in AI have addressed some of these barriers by enhancing the prediction of catalytic activities, improving stability, and enabling the design of targeted functionalities. Nevertheless, challenges remain, including data quality and accessibility issues, high‐dimensional data processing, model interpretability, and the transferability of AI models [204]. Addressing these challenges will be crucial for enhancing the reliability and scientific impact of AI‐assisted nanozyme research, while also expanding the application of AI across materials science, chemistry, and related fields.

## 7 Current Status of Nanozymes for Clinical Translation

### 7.1 Recent Progress of Nanozymes in Clinical Translation

Despite their promising therapeutic potential, most nanozymes remain at the experimental or preclinical stage. Their exceptional ability to regulate ROS and modulate immune responses facilitates the advancement of nanozymes toward clinical translation. Advances in nanomaterials have enabled the clinical approval of various nanoparticles [207]. Given these developments, nanozymes hold strong potential for clinical translation.

For example, ferumoxytol, an iron oxide nanoparticle exhibiting enzyme‐like properties, has been approved for the treatment of iron deficiency anemia (NCT01114217). Additionally, the robust POD‐like activity of ferumoxytol enables its application in the treatment of oral infections in humans (NCT03678012) [208]. Iron oxide nanoparticles are also employed as contrast agents for MRI. The M‐HFn nanozyme (Fe_3_O_4_ NPs), capable of targeting TfR1‐positive tumor tissues [181], provides a sensitive method for cancer imaging and holds potential for clinical translation.

Radiation dermatitis remains a persistent challenge for patients undergoing radiotherapy. Furthermore, effective clinical interventions for managing severe (Grade III) radiation‐induced skin injury remain limited. PB has been approved by the FDA for the treatment of radioactive exposure. The potent ROS‐scavenging capacity of PB‐based nanozymes shows therapeutic potential for ameliorating radiation dermatitis. Therefore, a novel PB‐based nanozyme combined with near‐infrared thermotherapy is being evaluated in patients with head and neck cancer (NCT07086638). This therapeutic approach offers a promising option for the treatment of severe Grade III radiation‐induced skin lesions.

Based on advances in nanozymes, several products are gradually entering clinical translation. Among them, cerium‐based nanozymes, owing to their unique multi‐enzyme activities and excellent biocompatibility, have become an important focus in nanozyme translational research. Cenyx Biotech, a South Korean nanomedicine company, has developed four cerium oxide‐based nanozymes for clinical trials. (i), CX213, a nanozyme‐P‐based anti‐inflammatory drug, has entered Phase 1 clinical trials for subarachnoid hemorrhage (SAH) and is also designed for the prevention of ALF. (ii) CX301, another nanozyme‐P‐based drug, is being developed for the emergency treatment of reperfusion‐failed stroke. In completed preclinical studies, CX301 demonstrated therapeutic efficacy in animal models of acute respiratory distress syndrome and trauma‐induced organ injuries. (iii), nanozyme‐D is a topical drug for the treatment of dermatitis and is currently undergoing preclinical efficacy evaluation. (iv), nanozyme‐E, a pH‐responsive oral drug, is developed for the treatment of ulcerative colitis and Crohn's disease. The remarkable ROS‐regulating potential of these nanozymes offers promising prospects for alleviating inflammatory conditions in both acute and chronic diseases.

### 7.2 Barriers to the Clinical Translation of Nanozymes

Despite their promising therapeutic potential in treating ROS‐related, inflammatory, and oxidative‐stress‐related diseases, a gap remains between preclinical research and clinical application. A key prerequisite for the clinical translation of nanozymes is the demonstration of excellent biocompatibility. Establishing a regulatory framework to address the ambiguous classification and missing evaluation standards requires large‐scale production criteria and legal supervision.

Biocompatibility represents the primary challenge for the clinical translation of nanozymes. Biocompatibility encompasses the direct cytotoxicity of nanozymes, their long‐term retention stability, and off‐target effects. In addition, the existing synthetic processes of many nanozymes remain complex. The lack of a standardized evaluation system constitutes a bottleneck for the clinical translation of nanozymes. To date, current regulations consider nanozymes as nanomedicines or nonbiological complex drugs [209]. Due to their unique biological properties, nanozymes should be regulated by a specific legal system. In this section, we focus on the biocompatibility of nanozymes and discuss strategies to address these challenges.

Within biological systems, both the stability and degradability of nanozymes are critical for ensuring their safe and effective application. Metal and metal oxide constitute the majority of nanozymes. The main toxicity mechanisms include ROS‐triggered apoptosis and ROS‐independent cell death. Iron oxide‐ and cerium‐based nanozymes show favorable biocompatibility and have entered clinical trials. However, other metal‐ and metal oxide‐based nanomaterials can induce harmful effects at the cellular and organ levels. Silver NPs are commonly considered safe nanomaterials. However, the oxidation states of silver inactivate the respiratory chain dehydrogenase, resulting in inflammation and apoptosis [210]. Systematic studies on the toxicity mechanisms of nanozymes facilitate their clinical translation.

Similarly, the biodistribution and retention time of nanozymes influence their potential toxicity to normal tissues, potentially leading to unexpected adverse effects. Some nanozymes induce transient adverse effects during the initial phase of treatment, which typically resolve within a few weeks. For example, a BSA@CeO/Fe^2+^ multifunctional nanozyme designed for breast tumor therapy increased the white blood cells and platelet counts on day 1 [211]. Similarly, a Cu‐based nanozyme induced abnormal hepatic enzyme levels, elevating alkaline phosphatase (ALP) and aspartate aminotransferase (AST) on day 2 [212]. The long‐term accumulation of 20 nm spherical AuNPs has been observed over a period of 120 days in mouse livers, spleens, and kidneys [213]. It is generally acknowledged that many nanozymes possess multiple enzyme‐like activities, which also raises concerns about triggering off‐target effects in healthy organs. A standard evaluation criterion remains necessary for long‐term safety assessment. Understanding the long‐term biocompatibility and biosafety of nanozymes will promote their potential clinical applications.

### 7.3 Regulations on Structural Features and Environmental Factors of Nanozymes for Minimizing Adverse Effects

To minimize adverse effects, stability, toxicity, and biocompatibility are primary considerations when selecting appropriate nanozymes, particularly metal and metal oxide‐based ones. High stability can prevent cell damage induced by the ion release. The toxicity of nanozymes is affected by their structural features, such as size, morphology, and surface chemical properties. For many nanozymes, their toxicity is closely correlated with the administered dosage. For example, a low dosage of Arctium lappa root phenolic nanofibers (ALPRF‐NF) exhibits antioxidant therapeutic effects in models of polycystic ovary syndrome. However, excessive ROS scavenging disturbs the normal functions of the ovaries [214]. The optimal dosage is not only determined by the therapeutic goal but also balances potential adverse effects. Retention time is also a key challenge in nanozyme applications. Depending on the therapeutic goals for acute versus chronic diseases, different strategies are employed. The size modulation (< 5.5 nm) of nanozymes can significantly reduce the retention time by renal elimination. To achieve extended circulation, surface modification such as PEGylation can prolong the half‐life of nanozymes. Targeting strategies, such as mannose modification for KCs and GalNAc conjugation for hepatocyte delivery, can effectively reduce non‐specific accumulation in extrahepatic organs. These strategies provide effective solutions to overcome long‐term retention and off‐target challenges, thereby facilitating the clinical translation of nanozymes.

As mentioned above, environmental factors such as pH and temperature constrain the catalytic reactions of nanozymes. Regulation of environmental pH and enhancement of nanozyme catalytic performance can overcome these limitations for therapeutic applications. However, these restrictions can also serve as a useful strategy to minimize off‐target enzyme‐like reactions. Cold‐activated nanozymes exhibit promising potential for spatially restricting catalytic activity to specific tissues via temperature control [77]. Local pH regulation can precisely enhance the catalytic performance of nanozymes at targeted organs. The liver is a major site of glucose production. Thus, the GOx‐mediated pH‐lowering strategy can be effectively combined with POD‐like nanozymes [62], due to their insufficient catalytic performance in other healthy tissues.

Clinical translation demands nanozymes possessing outstanding biocompatibility and long‐term stability under physiological conditions. Regulations of intrinsic properties of nanozymes, such as size and surface modification, have a profound effect on their in vivo fate. pH and temperature limitations can act as both constraints and opportunities. Reduced toxicity and controllable catalytic activity help overcome key barriers, facilitating the clinical translation of nanozymes.

## 8 Conclusion and Outlook

The liver plays a dominant role in lipid metabolism, redox reactions, and the maintenance of physiological functions. These processes include numerous enzymatic reactions. Preliminary studies indicate that nanozymes show promise for treating liver‐associated diseases by mimicking enzyme‐like reactions. However, the existing mismatch between optimal catalytic reactions and pathological conditions limits the therapeutic efficacy of nanozymes in liver diseases. To address this issue, strategies involving the regulation of nanozyme structural features and the remodeling of environmental conditions are being explored. Nanozymes are emerging therapeutic tools with multi‐enzyme‐mimicking catalytic activities, capable of scavenging ROS, regulating oxidative stress, degrading biomacromolecules, and modulating cellular pathways. Nanozymes have shown efficacy in modulating metabolic dysfunction, mitigating ALF/ALI, promoting hepatocyte regeneration, attenuating HIRI, inhibiting HCC progression, and treating other liver‐associated diseases. Some of these nanozymes are advancing toward clinical translation and demonstrate significant potential in liver disease therapy.

Despite encouraging advances, several challenges still limit nanozymes’ clinical translation. The requirements for long‐term biocompatibility, along with the lack of scale‐up production evaluation and legal supervision, restrict the broad clinical application of nanozymes. Among these, the biocompatibility of nanozymes should be prioritized by researchers in the early stages. Overcoming other barriers requires assistance from companies and government agencies. Nanozymes with different compositions or enzymatic activities show good biocompatibility both in vitro and in vivo with basic toxicological endpoints [215]. However, realizing long‐term biocompatibility demands high stability, proper retention time, and minimal off‐target effects, which are key challenges hindering their clinical applications. Regulations on structural features directly affect the toxicity, targeted organ accumulation, and elimination pathways. Environmental factors, such as pH and temperature, can limit the catalytic performance of nanozymes. Therefore, specific environmental regulation at the diseased area minimizes the off‐target effects in healthy tissues.

From a translational perspective, the future development of nanozymes should address the following considerations: First, an extensive range of enzymatic reactions is necessary. The novel definition allows for the difference between nanozymes and the corresponding enzymes [6]. The catalytic activities should not be restricted to the existing framework of oxidoreductases and hydrolases. In addition, natural polyphenols, when combined with nanozymes, may provide enhanced inhibition of ferroptosis during ALI [107], providing a compelling rationale for developing novel nanozyme hybrids. Nanozymes can also be regarded as a combination therapy, complementing the traditional surgery, radiotherapy, and immunotherapy. Nanozymes exhibit potential both for collaborative treatments and for reducing the adverse effects induced by other therapies.

Moreover, there is a promising prospect for the design of controllable nanozymes. The lack of precise control limits the therapeutic potential of nanozymes [216]. Heat, light, and ultrasound are desirable triggers for the development of intelligent nanozymes. These external stimuli can be precisely applied to the diseased region, minimizing the off‐target effects caused by non‐specific nanozyme delivery. Substrate specificity determines the catalytic reactions of nanozymes. Under diseased conditions, increased glucose (T2D) and H_2_O_2_ (HCC) levels offer opportunities for substrate‐dependent controllable catalytic therapy. These substrates allow the activation of GOx catalytic reactions at the diseased area. Furthermore, catalytic reactions alter the environmental factors by H^+^ production, thus activating other nanozymes’ catalytic performance [62]. Of note, intelligent nanozymes are also expected to modulate their catalytic performance during different disease stages. For example, during the early stage of wound healing, nanozymes with pro‐oxidant properties prevent bacterial infection. In later stages, the antioxidant activity of nanozymes facilitates tissue repair [217]. Intelligent nanozymes satisfy the growing demands for precision medicine and solve challenges faced by traditional nanozymes.

In conclusion, this review provides a comprehensive overview of recent advances in nanozymes for biomedical applications, especially in the treatment of liver diseases. Nanozymes have substantial potential for liver disease therapy by combining ROS regulation, enzyme replacement, and microenvironment modulation. However, a gap remains between the ideal catalytic conditions and the pathological environment in the liver. Regulation of structural features or modification of environmental factors restores the catalytic performance of nanozymes under diseased conditions. To advance clinical translation, future developments should aim to integrate targeted delivery, controllable catalytic activity, and comprehensive safety evaluation to maximize therapeutic efficacy.

## Funding

National Key Research and Development Program of China (2024YFA0918600), National Natural Science Foundation of China (82325029, U22A20156, W2441022, 32171379, China), Science and technology research project of the Education Department of Jilin Province (JJKH20231222KJ, JJKH20261512KJ and JJKH20261427KJ), and the Fundamental Research Funds for the Central Universities, Jilin University (China).

## Ethics Approval and Patient Consent

Ethical approval and patient consent were not required as this is a review article based on the analysis of previously published literature. This study does not involve any clinical trials, and thus, clinical study registration is not applicable.

## Conflicts of Interest

The authors declare no conflicts of interest.

## Supporting information




**Supporting File**: advs74590‐sup‐0001‐SuppMat.docx.

## Data Availability

Data sharing is not applicable to this article as no new data were created or analyzed in this study.
