# Supplementary material for: Nanozymes for Liver Disease Therapy: Advances in Catalytic Activity, Targeting Strategies, and Clinical Translation
Source: Adv Sci (Weinh). 2026 Feb 28;13(20):e74590. doi: 10.1002/advs.74590 (PMC13067777; doi:10.1002/advs.74590)
Supplement: Supplementary file 1 — Supporting File: advs74590‐sup‐0001‐SuppMat.docx. [file ADVS-13-e74590-s001.docx]

Supporting information

Nanozymes for Liver Disease Therapy: Advances in Catalytic Activity, Targeting Strategies, and Clinical Translation

Xiandi Meng, Ge Zhu, Siyu Sun, Wenbo Yao, Yuning Zhang, Yong-Guang Yang, and Tianmeng, Sun*

X. Meng, G. Zhu, S. Sun, W. Yao, Y. Zhang, Y-G. Yang, T. Sun

Key Laboratory of Organ Regeneration and Transplantation of Ministry of Education, Institute of Immunology, The First Hospital, Jilin University, Changchun, Jilin, China;

National-local Joint Engineering Laboratory of Animal Models for Human Diseases, Changchun, Jilin, China;

E-mail: tsun41@jlu.edu.cn

G. Zhu

Cancer Center, The First Hospital of Jilin University, Changchun, Jilin, 130021, China;

Y-G. Yang, T. Sun
International Center of Future Science, Jilin University, Changchun, Jilin, China;

State Key Laboratory of Supramolecular Structure and Materials, Jilin University, Changchun, Jilin, China.

Keywords: nanozymes, liver disease, redox reaction, hydrolases, ROS regulation

Box 1. Composition of nanozymes.

Metal nanozymes. Metal-based nanozymes, as the earliest discovered, have been widely applied in various biomedical fields with remarkable therapeutic effects. Although the majority are considered biologically inert, the unique structures and electronic properties of nanoscale metallic materials have been utilized in many fields^[1]^. Due to their biocompatibility, ease of scalable production, and controllable activity, Au, Ag, Pt, and Pd are expected to be excellent nanomaterials for nanozymes over the past decades. Metal Oxide-Based Nanozymes. Metal oxide nanozymes usually exhibit high surface energy and surface-to-volume ratio, possessing promising artificial enzyme properties^[2]^. Widely utilized metal oxide nanozymes like Fe_3_O_4_, CeO_2_, and Mn_3_O_4_ nanomaterials have been proved with multi-enzyme-like activities^[3, 4]^. Compared to noble metal nanozymes, oxide-based nanozymes show lower biological toxicity and higher accumulation in organs. However, under physiological conditions, unmodified metal oxide nanozymes may remain stable and generate harmful free radicals^[5]^. Metal-organic frameworks (MOFs). MOFs are porous solid materials characterized by well-defined coordination networks, mesoporous structure, and tunable porosity^[6]^. Due to their excellent catalytic properties, MOFs have been widely used in antibacterial, biosensing, bioimaging and other biological applications. With different combinations of metal nodes and organic ligands, more than 20,000 MOFs have been synthesized. Based on the chemical characteristics, MOF-based nanozymes can be categorized into three types: 1) pristine MOFs (self-assembled metal nodes and organic ligands), 2) MOF-based composites (MOF/nanoparticles, MOF/polymer, MOF/graphene and MOF/carbon nanotube), and 3) MOF-based derivatives (carbon material, metal-carbon material, single-atom material and metal compound). Carbon-based Nanozymes. Carbon nanomaterials (CNMs), including fullerenes, carbon nanotubes (CNTs), graphene, graphene oxide (GO), carbon quantum dots (CQDs), and graphene quantum dots (GQDs), possess excellent biological catalytic activity. There is a growing interest in CNMs due to their metal-free catalytic ability in biochemical reactions. Generally, CNMs exhibit SOD- and POD-like catalytic activities in various research fields, including biosensing, photocatalysis, and biomedical theranostics^[7-9]^.

**Box 2.** The internal structural features of nanozymes.

The catalytic performance of nanomaterials is closely associated with the size of the nanozymes. In most cases, the catalytic performance gradually decreases as the size of nanozymes increases. One possible reason is that a higher surface-to-volume ratio is easily achieved, facilitating interactions with corresponding substrates. For example, 13, 20, 30, and 50 nm Au NPs were synthesized by Fan et al. to demonstrate the reaction rates under the same conditions. The GOx-like catalytic activity of AuNPs decreases as the size increases^[10]^. Interestingly, in some cases, nanozymes with larger size achieve better catalytic properties. Oligonucleotide capped 2.9 nm Pt nanozymes exhibited higher POD-like activity than 1.8 nm Pt. The reason is 2.9 nm Pt nanozymes contained more metallic Pt^0^ for enzyme-like functions^[11]^.

The morphology of nanomaterials also determines their catalytic properties. The shapes of nanozymes can be divided into a variety of classes. Commonly utilized nanozymes include rods, spheres, cubes, polyhedra, nanoflowers, flakes, and hexagonal plates. Mugesh and co-workers measured the CAT-, GPx-like and SOD-like activities of differently shaped Mn_3_O_4_ nanozymes. In their results, only the flower-shaped Mn_3_O_4_ nanozymes exhibited all three catalytic activities, while other morphologies only performed SOD-like activities^[12]^. Yin’s group designed two kinds of Pd nanozymes, nanocubes and octahedrons, to evaluate their SOD- and CAT-like activities. In another work reported by Pathak and colleagues, the POD-like catalytic ability was compared between octahedron and spherical shaped Fe_3_O_4_ nanoparticles. The results indicated that octahedron-shaped Fe_3_O_4_ possessed higher catalytic ability^[13]^.

During nanozyme-related studies, surface modification is the most commonly applied approach to enhance catalytic abilities, including functional groups, coating thickness, and surface charges. These surface modifications improve the interactions for substrate. Au nanozymes with different surface modifications can exhibit various catalytic properties. Mesoporous silica-modified Au nanozymes performed GOx-like ability, while POD-like activity was exhibited when citrate-modified^[14]^. Amino-terminated Au NPs (AuNCs-NH_2_) can selectively inhibit the POD-like but retain the CAT-like catalytic activities^[15]^. Inspired by natural enzymes, the affinity of histidine-modified Fe_3_O_4_ with H_2_O_2_ was significantly improved, resulting in a 20-fold increase in POD-like catalytic efficiency^[16]^.

The catalytic activities of nanozymes can generally be regulated by adjusting their composition. Increasing one nanomaterial or reducing another element can change the catalytic activities. One widespread application is growing more active nanomaterials on less active ones to enhance catalytic efficiency. For example, coating Ir layers on Pd nanocubes enhanced catalytic efficiency by at least 20- to 400-fold compared to previous designs^[17]^. Wei’s group also designed multi-functional Au@Pt nanozymes. A higher catalytic activity can be achieved with the increased Pt shell on the Au core, shortening the time for H_2_O_2_ detection and enhancing sensitivity^[18]^.

**Abbreviations**

| Reactive oxygen species | ROS |
| --- | --- |
| Artificial intelligence | AI |
| Gold nanoparticles | AuNPs |
| Peroxidase | POD |
| Non-alcoholic fatty liver disease | NAFLD |
| Hepatic ischemia-reperfusion injury | HIRI |
| Hepatocellular carcinoma | HCC |
| Metabolic dysfunction**-**associated steatohepatitis | MASH |
| Kupffer cells | KCs |
| Hepatic stellate cells | HSCs |
| Oxidase | OXD |
| Glucose oxidase | GOx |
| Graphene Oxide | GO |
| Prussian blue | PB |
| Ce_0.5_Zr_0.2_Nb_0.15_Ta_0.1_Hf_0.05_O_x_ | CZNTHO |
| Bovine serum albumin | BSA |
| Phosphodiesterase | PDE |
| Cyclic guanosine monophosphate | cGMP |
| Cyclic adenosine monophosphate | cAMP |
| Glutathione | GSH |
| Glomerular endothelial cells | GECs |
| Reticuloendothelial system | RES |
| Apolipoprotein E | apoE |
| Asialoglycoprotein receptor | ASGPR |
| N-acetylgalactosamine | GalNAc |
| 9-fluorenylmethoxycarbonyl-modified diphenylalanine | Fmoc-FF |
| Poly(acrylic acid) | PAA |
| Poly(ethylene imine) | PEI |
| 3,3',5,5'-Tetramethylbenzidine | TMB |
| Aspartic acid | asp |
| Hollow Prussian blue nanocubes | hPBNCs |
| Polyamidoamine dendrimer | PAMAM |
| Tumor microenvironment | TME |
| Glutathione oxidase | GSH-OXD |
| Single-atom nanozymes | Sazymes |
| Mild photothermal therapy | mPTT |
| Ethylenediaminetetraacetic acid disodium salt | EDTA-2Na |
| Malachite green carbinol base | MGCB |
| Acute liver failure | ALF |
| Metabolic dysfunction-associated fatty liver disease | MAFLD |
| Nonalcoholic steatohepatitis | NASH |
| Food and Drug Administration | FDA |
| Two-dimensional | 2D |
| Tumor necrosis factor-α | TNF-α |
| Interleukin-6 | IL-6 |
| Interleukin-1β | IL-1β |
| Nanocrystalline cerium dioxide | nCeO_2_ |
| Hollow CeO_2_ | H-CeO_2_ |
| Resveratrol | Res |
| N-acetylcysteine | NAC |
| Gold nanoclusters | Au NCs |
| Hepatocyte-like cells | HLCs |
| Acetaminophen | APAP |
| Two-pronged black phosphorus/Ceria nanozyme | TBP@CeO2 |
| Extracellular matrix | ECM |
| Hepatitis B virus | HBV |
| Hepatitis C virus | HCV |
| Carbon nanodots | CNDs |
| Microneedle | MN |
| Soy protein isolate | SPI |
| Polyvinyl alcohol | PVA |
| Stem cell secretome nanoparticles | SecNPs |
| Pt-based nanozymes | PtNZs |
| Neutral protease | NPr |
| Near-infrared | NIR |
| Hyaluronic acid | HA |
| Nilotinib | NIL |
| Cerium oxide nanoparticles | CeO2 NPs |
| HA-targeting liposome delivery system | HCOL |
| Oleanolic acid | OA |
| Ascorbic acid-oxidase | AAO |
| Pt@Pd-based hedgehogs | PPNHs |
| Liquiritigenin | LQ |
| Lysyl oxidase-like 2 | LOXL2 |
| Carvedilol | CAR |
| Platinum nanoparticles | Pt NPs |
| Mesoporous organosilica nanoparticles | MONs |
| Phenylboronic acid pinacol ester | PBAP |
| Adeno-associated virus serotype 8 | AAV8 |
| Tannic acid | TA |
| Trojan horse-like biohybrid nanozyme | THBN |
| Carbon dots | C-dots |
| Magnetic resonance imaging | MRI |
| Immune-checkpoint inhibitors | ICIs |
| Regulated cell death | RCD |
| Radiofrequency ablation | RFA |
| Incomplete radiofrequency ablation | iRFA |
| Radiofrequency dynamic therapy | RFDT |
| Low-intensity radiofrequency | LIRF |
| Interleukin-2 | IL-2 |
| Interleukin-12p70 | IL-12p70 |
| Interferon-γ | IFN-γ |
| Photothermal therapy | PTT |
| Sonodynamic therapy | SDT |
| Magnetoferritin | M-HFn |
| Transferrin receptor 1 | TfR1 |
| Direct acting antivirals | DAAs |
| RNA interference | RNAi |
| Type 2 diabetes | T2D |
| Lactobionic acid | LA |
| Disulfiram | DSF |
| Nuclear factor-κB | NFκB |
| β-lactoglobulin | BLG |
| Subarachnoid hemorrhage | SAH |
| Alkaline phosphatase | ALP |
| Aspartate aminotransferase | AST |
| Arctium lappa root phenolic nanofibers | ALPRF-NF |

| Reactive oxygen species | ROS |
| --- | --- |
| Artificial intelligence | AI |
| Gold nanoparticles | AuNPs |
| Peroxidase | POD |
| Non-alcoholic fatty liver disease | NAFLD |
| Hepatic ischemia-reperfusion injury | HIRI |
| Hepatocellular carcinoma | HCC |
| Metabolic dysfunction-associated steatohepatitis | MASH |
| Kupffer cells | KCs |
| Hepatic stellate cells | HSCs |
| Oxidase | OXD |
| Glucose oxidase | GOx |
| Graphene Oxide | GO |
| Reduced Graphene Oxide | rGO |
| Prussian blue | PB |
| Ce0.5Zr0.2Nb0.15Ta0.1Hf0.05Ox | CZNTHO |
| Bovine serum albumin | BSA |
| Phosphodiesterase | PDE |
| Cyclic guanosine monophosphate | cGMP |
| Cyclic adenosine monophosphate | cAMP |
| Glutathione | GSH |
| Glomerular endothelial cells | GECs |
| Reticuloendothelial system | RES |
| Apolipoprotein E | apoE |
| Asialoglycoprotein receptor | ASGPR |
| N-acetylgalactosamine | GalNAc |
| 9-fluorenylmethoxycarbonyl-modified diphenylalanine | Fmoc-FF |
| Poly(acrylic acid) | PAA |
| Poly(ethylene imine) | PEI |
| 3,3',5,5'-Tetramethylbenzidine | TMB |
| Aspartic acid | asp |
| Hollow Prussian blue nanocubes | hPBNCs |
| Polyamidoamine dendrimer | PAMAM |
| Tumor microenvironment | TME |
| Glutathione oxidase | GSH-OXD |
| Single-atom nanozymes | Sazymes |
| Mild photothermal therapy | mPTT |
| Ethylenediaminetetraacetic acid disodium salt | EDTA-2Na |
| Malachite green carbinol base | MGCB |
| Acute liver failure | ALF |
| Metabolic dysfunction-associated fatty liver disease | MAFLD |
| Nonalcoholic steatohepatitis | NASH |
| Food and Drug Administration | FDA |
| Two-dimensional | 2D |
| Tumor necrosis factor-α | TNF-α |
| Interleukin-6 | IL-6 |
| Interleukin-1β | IL-1β |
| Nanocrystalline cerium dioxide | nCeO2 |
| Hollow CeO2 | H-CeO2 |
| Resveratrol | Res |
| N-acetylcysteine | NAC |
| Gold nanoclusters | Au NCs |
| Hepatocyte-like cells | HLCs |
| Acetaminophen | APAP |
| Two-pronged black phosphorus/Ceria nanozyme | TBP@CeO2 |
| Extracellular matrix | ECM |
| Hepatitis B virus | HBV |
| Hepatitis C virus | HCV |
| Carbon nanodots | CNDs |
| Microneedle | MN |
| Soy protein isolate | SPI |
| Polyvinyl alcohol | PVA |
| Stem cell secretome nanoparticles | SecNPs |
| Pt-based nanozymes | PtNZs |
| Neutral protease | NPr |
| Near-infrared | NIR |
| Hyaluronic acid | HA |
| Nilotinib | NIL |
| Cerium oxide nanoparticles | CeO2 NPs |
| HA-targeting liposome delivery system | HCOL |
| Oleanolic acid | OA |
| Ascorbic acid-oxidase | AAO |
| Pt@Pd-based hedgehogs | PPNHs |
| Liquiritigenin | LQ |
| Lysyl oxidase-like 2 | LOXL2 |
| Carvedilol | CAR |
| Platinum nanoparticles | Pt NPs |
| Mesoporous organosilica nanoparticles | MONs |
| Phenylboronic acid pinacol ester | PBAP |
| Adeno-associated virus serotype 8 | AAV8 |
| Tannic acid | TA |
| Trojan horse-like biohybrid nanozyme | THBN |
| Carbon dots | C-dots |
| Magnetic resonance imaging | MRI |
| Immune-checkpoint inhibitors | ICIs |
| Regulated cell death | RCD |
| Radiofrequency ablation | RFA |
| Incomplete radiofrequency ablation | iRFA |
| Radiofrequency dynamic therapy | RFDT |
| Low-intensity radiofrequency | LIRF |
| Interleukin-2 | IL-2 |
| Interleukin-12p70 | IL-12p70 |
| Interferon-γ | IFN-γ |
| Photothermal therapy | PTT |
| Sonodynamic therapy | SDT |
| Magnetoferritin | M-HFn |
| Transferrin receptor 1 | TfR1 |
| Direct acting antivirals | DAAs |
| RNA interference | RNAi |
| Type 2 diabetes | T2D |
| Lactobionic acid | LA |
| Disulfiram | DSF |
| Nuclear factor-κB | NFκB |
| β-lactoglobulin | BLG |
| Subarachnoid hemorrhage | SAH |
| Alkaline phosphatase | ALP |
| Aspartate aminotransferase | AST |
| Arctium lappa root phenolic nanofibers | ALPRF-NF |

**References**

[1] D. Xu, L. Wu, H. Yao, L. Zhao, Catalase-Like Nanozymes: Classification, Catalytic Mechanisms, and Their Applications, Small, 18 (2022) e2203400.

[2] R. Narayanan, M.A. El-Sayed, Catalysis with transition metal nanoparticles in colloidal solution: nanoparticle shape dependence and stability, J Phys Chem B, 109 (2005) 12663-12676.

[3] L. Gao, J. Zhuang, L. Nie, J. Zhang, Y. Zhang, N. Gu, T. Wang, J. Feng, D. Yang, S. Perrett, X. Yan, Intrinsic peroxidase-like activity of ferromagnetic nanoparticles, Nat Nanotechnol, 2 (2007) 577-583.

[4] J. Golchin, K. Golchin, N. Alidadian, S. Ghaderi, S. Eslamkhah, M. Eslamkhah, A. Akbarzadeh, Nanozyme applications in biology and medicine: an overview, Artif Cell Nanomed B, 45 (2017) 1069-1076.

[5] S.V. Salihov, Y.A. Ivanenkov, S.P. Krechetov, M.S. Veselov, N.V. Sviridenkova, A.G. Savchenko, N.L. Klyachko, Y.I. Golovin, N.V. Chufarova, E.K. Beloglazkina, A.G. Majouga, Recent advances in the synthesis of FeO@AU core/shell nanoparticles, J Magn Magn Mater, 394 (2015) 173-178.

[6] H.C. Zhou, J.R. Long, O.M. Yaghi, Introduction to metal-organic frameworks, Chem Rev, 112 (2012) 673-674.

[7] Y.Q. Sun, H.Y. Qin, X. Geng, R. Yang, L.B. Qu, A.N. Kani, Z.H. Li, Rational Design of Far-Red to Near-Infrared Emitting Carbon Dots for Ultrafast Lysosomal Polarity Imaging, Acs Appl Mater Inter, 12 (2020) 31738-31744.

[8] M.L. Liu, B.B. Chen, C.M. Li, C.Z. Huang, Carbon dots: synthesis, formation mechanism, fluorescence origin and sensing applications, Green Chem, 21 (2019) 449-471.

[9] X. Geng, Y.Q. Sun, Z.H. Li, R. Yang, Y.M. Zhao, Y.F. Guo, J.J. Xu, F.T. Li, Y. Wang, S.Y. Lu, L.B. Qu, Retrosynthesis of Tunable Fluorescent Carbon Dots for Precise Long-Term Mitochondrial Tracking, Small, 15 (2019) 1901517.

[10] W. Luo, C. Zhu, S. Su, D. Li, Y. He, Q. Huang, C. Fan, Self-catalyzed, self-limiting growth of glucose oxidase-mimicking gold nanoparticles, ACS Nano, 4 (2010) 7451-7458.

[11] Y. Fu, X.Y. Zhao, J.L. Zhang, W. Li, DNA-Based Platinum Nanozymes for Peroxidase Mimetics, J Phys Chem C, 118 (2014) 18116-18125.

[12] N. Singh, M.A. Savanur, S. Srivastava, P. D'Silva, G. Mugesh, A Redox Modulatory Mn(3) O(4) Nanozyme with Multi-Enzyme Activity Provides Efficient Cytoprotection to Human Cells in a Parkinson's Disease Model, Angew Chem Int Ed Engl, 56 (2017) 14267-14271.

[13] N. Puvvada, P.K. Panigrahi, D. Mandal, A. Pathak, Shape dependent peroxidase mimetic activity towards oxidation of pyrogallol by HO, Rsc Adv, 2 (2012) 3270-3273.

[14] Y. Lin, Z. Li, Z. Chen, J. Ren, X. Qu, Mesoporous silica-encapsulated gold nanoparticles as artificial enzymes for self-activated cascade catalysis, Biomaterials, 34 (2013) 2600-2610.

[15] C.P. Liu, T.H. Wu, Y.L. Lin, C.Y. Liu, S. Wang, S.Y. Lin, Tailoring Enzyme-Like Activities of Gold Nanoclusters by Polymeric Tertiary Amines for Protecting Neurons Against Oxidative Stress, Small, 12 (2016) 4127-4135.

[16] K. Fan, H. Wang, J. Xi, Q. Liu, X. Meng, D. Duan, L. Gao, X. Yan, Optimization of Fe(3)O(4) nanozyme activity via single amino acid modification mimicking an enzyme active site, Chem Commun (Camb), 53 (2016) 424-427.

[17] X. Xia, J. Zhang, N. Lu, M.J. Kim, K. Ghale, Y. Xu, E. McKenzie, J. Liu, H. Ye, Pd-Ir Core-Shell Nanocubes: A Type of Highly Efficient and Versatile Peroxidase Mimic, ACS Nano, 9 (2015) 9994-10004.

[18] J. Wu, K. Qin, D. Yuan, J. Tan, L. Qin, X. Zhang, H. Wei, Rational Design of Au@Pt Multibranched Nanostructures as Bifunctional Nanozymes, ACS Appl Mater Interfaces, 10 (2018) 12954-12959.
